# Elucidating the molecular mechanisms underlying the induction of autophagy by antidepressant-like substances in C57BL/6J mouse testis model upon LPS challenge

**DOI:** 10.1186/s12964-023-01270-6

**Published:** 2023-09-21

**Authors:** Przemysław Sołek, Ewelina Czechowska, Magdalena Sowa-Kućma, Katarzyna Stachowicz, Piotr Kaczka, Anna Tabęcka-Łonczyńska

**Affiliations:** 1 Department of Biopharmacy, Medical University of Lublin, 4a Chodźki, 20-093 Lublin, Poland; 2 Department of Human Physiology, Institute of Medical Sciences, Medical College of Rzeszow University, 2a Kopisto, 35-959 Rzeszow, Poland; 3 Maj Institute of Pharmacology Polish Academy of Sciences, 12 Smetna, 31-343 Krakow, Poland; 4 PRO-NOO-BIOTICS Sp. z o.o., 39 Warszawska, 35-205 Rzeszow, Poland; 5 Department of Biotechnology and Cell Biology, Medical College, University of Information Technology and Management in Rzeszow, 2 Sucharskiego, 35-225 Rzeszow, Poland

**Keywords:** Antidepressant-like substances, Autophagy, Depression, Endoplasmic reticulum, Golgi apparatus, Testis

## Abstract

The treatment of depression with pharmaceuticals is associated with many adverse side effects, including male fertility problems. The precise mechanisms by which these agents affect testicular cells remain largely unknown, but they are believed to induce cellular stress, which is sensed by the endoplasmic reticulum (ER) and the Golgi apparatus. These organelles are responsible for maintaining cellular homeostasis and regulating signal pathways that lead to autophagy or apoptosis. Therefore, in this study, we aimed to investigate the autophagy, ER, and Golgi stress-related pathways in mouse testis following treatment with antidepressant-like substances (ALS) and ALS combined with lipopolysaccharide (LPS). We found that most ALS and activated proteins are associated with the induction of apoptosis. However, when imipramine (IMI) was combined with NS-398 (a cyclooxygenase-2 inhibitor) after LPS administration, we observed a marked increase in the BECLIN1, Bcl-2, ATG16L, and LC3 expression, which are marker proteins of autophagosome formation. The expression of the *BECN1* and *ATG16L* genes was also high compared to the control, indicating the induction of autophagy processes that may potentially protect mouse testicular cells from death and regulate metabolism in the testis. Our findings may provide a better understanding of the stress-related effects of specific ALS on the testis.

**Graphical Abstract:**

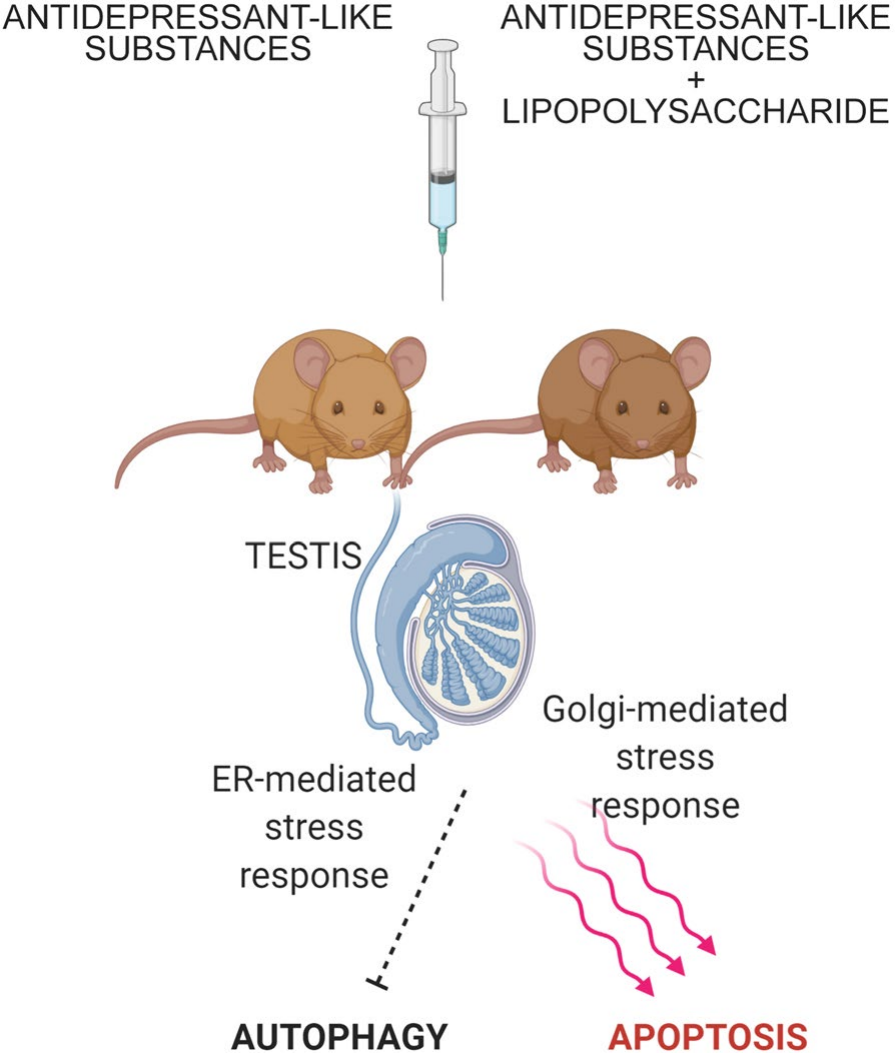

Video Abstract

**Supplementary Information:**

The online version contains supplementary material available at 10.1186/s12964-023-01270-6.

## Highlights

• ALS and ALS with LPS mostly generate apoptosis

• IMI with NS-398 with LPS induce the expression of proteins involved in autophagy

• IMI with NS-398 with LPS increase the expression of genes involved in autophagy

• IMI with NS-398 with LPS potentially may protect mouse testicular cells from death and regulate metabolism in testis by autophagy process.

**Supplementary Information:**

The online version contains supplementary material available at 10.1186/s12964-023-01270-6.

## Introduction

The growing prevalence of depression underscores the need for novel therapies that offer improved treatment outcomes while mitigating the adverse effects associated with pharmacological interventions. The use of antidepressants has been linked to drug-induced toxicity, which can result in temporary or permanent reductions in fertility [1, 2]. However, the molecular mechanisms that underlie the effects of antidepressants on testicular cells remain poorly understood.

Cellular stress may arise from both internal and external factors, leading to processes that either maintain cellular homeostasis or trigger cell death [3]. Physiological and pathological stressors can cause protein folding disorders and the accumulation of misfolded proteins within cells. In response, cells activate the unfolded protein response (UPR) to mitigate stress. Chronic exposure to pharmaceuticals can induce stress in cells, with the endoplasmic reticulum (ER) and Golgi apparatus serving as intracellular precision sensors [4, 5]. Such stress may lead to the diversion of cells into either autophagy or apoptosis [6–9]. The ER and Golgi complex are part of the internal endomembrane system and various substances are constantly transported between these organelles [10]. It has been shown that disturbances in ER and Golgi apparatus functions can lead to disturbances in the transport of proteins needed for autophagy [11, 12].

Autophagy is the evolutionarily conserved process, that can prevent cell death in specified biological situations, but it is sometimes an alternative route to cell death also [13]. The proper testicular development is regulated by autophagy, which may indicate a high involvement of this process [14]. Thus, autophagy is a critical lysosomal pathway for cell survival [15]. Various signal transduction mechanisms are implicated in the regulation of macroautophagy, in response to various extra- and intracellular stimuli. Protein kinase A (PKA), 5’-AMP-activated protein kinase (AMPK), and mammalian target of rapamycin complex 1 (mTORC1) are the three primary kinases implicated in the regulation of autophagy.

Programmed cell death or apoptosis are a complex, multi-level processes [16], which are closely regulated and interdependent with other cellular pathways. The UPR plays a crucial role in determining whether a cell will undergo apoptosis when cellular stress surpasses a threshold level [17].

The testis serves as a site for continuous production of male reproductive cells. In this study, we aim to elucidate the step-by-step transduction signaling processes involving the ER, Golgi apparatus, and autophagy in the testicular tissue of male mice following treatment with antidepressant-like substances (ALS). The administration of LPS served as the stress-inducing stimulus that triggered depressive symptoms in mice [18–21]. Perturbations in these pathways can interfere with cell homeostasis, thereby directing cells towards apoptosis or autophagy, and could potentially impact spermatogenesis regulation by influencing cholesterol reuptake and testosterone synthesis [14, 22]. A comprehensive understanding of the molecular mechanisms underpinning these pathways in this critical reproductive organ may have important implications for safeguarding male fertility, reducing adverse side effects in the male reproductive system, and ultimately contributing towards the mitigation of infertility in humans [22].

## Materials and methods

### Animals

Male C57BL/6J mice (6–10 week old) (*n* = 7 for each group) were housed under constant laboratory conditions, during the light period (8:00–16:00), and with constant access to food and water. The procedures were carried out based on the National Institutes of Health Animal Care and Use Committee and were approved by the Ethics Committee of the Maj Institute of Pharmacology, Polish Academy of Sciences in Krakow (Protocol No 178/2017).

### Drug treatment and tissue collection

The following substances were used: 3-[(2-methyl-1,3-tiazol-4-yl)ethynyl]-pyridine (MTEP; Tocris Cookson ltd., Bristol, UK); *N*-[2-(Cyclohexyloxy)-4-nitrophenyl]methanesulfonamide (NS-398, Abcam Biochemicals, UK); Imipramine (hydrochloride, Sigma-Aldrich, Saint Louis, MO, USA), LPS serotype 0127:B8 (Sigma-Aldrich, Saint Louis, MO, USA). NS-398 (3 mg/kg) was dissolved in 10% DMSO; Imipramine (10 mg/kg) and LPS (0.83 mg/kg) were used as aqueous solution; MTEP (1 mg/kg) was prepared as 1% aqueous solution in Tween 80. 10% DMSO was used for vehicle group injections. These substances were injected intraperitoneally (*i.p.)* once a day at the same time for 14 consecutive days. Separate groups of mice were used for experiments using LPS serotype 0127:B8 (Sigma-Aldrich, Saint Louis, MO, USA)(0.83 mg/kg), and the substances mentioned above with LPS. These animals had an additionally enriched environment to hide during symptoms caused by the use of substances and LPS. Proper control and LPS-treated groups were provided, while the last LPS treatment occurred 24 h before the last injection of substances used. Then, 24 h after the last scheduled administration of drugs, the animals were decapitated and the samples of testicular tissues were freshly dissected and stored at − 80 °C until the start of the planned analyses.

### Antibodies

The following primary antibodies were used in this study: - Reference protein: anti-β-actin (1:10,000; #PA1-16,889, RRID: AB_568434) (Thermo Scientific, Saint Louis, MO, USA);

- Autophagy pathway: anti-mTOR (1:1000; #PA5-34,663, RRID: AB_2552015), anti-ULK1 (1:1000; #PA5-26,126, RRID: AB_2543626), anti-ATG13 (1:1000; #PA5-26923, RRID: AB_2544423), anti-BECLIN1 (1:1000; #PA1-16857, RRID: AB_568459), anti-ATG14 (1:1000; #PA5-34972, RRID: AB_2552321), anti-ATG5 (1:1000; # PA5-23186, RRID: AB_2540712), anti-ATG16L1 (1:1000; #PA1-46307, RRID: AB_2059398), anti-LC3A/B (1:1000, #PA1-16931, RRID: AB_2137583) (Thermo Scientific, Saint Louis, MO, USA) or anti-BCl2 (1:500; #sc-7382, RRID:AB_626736; Santa Cruz, USA);
- Golgi stress pathway: anti-TFE3 (1:500; #PA5-54909, RRID: AB_2648409), anti-HSP47 (1:1000; #PA5-14254,RRID: AB_2285672), anti-CREB34L (1:750; #PA5-18028, RRID: AB_10982190), anti-ARF4 (1:1000;#PA5-37841, RRID: AB_2554449), anti-SIAT4A (1:2000; #PA5-21721, RRID: AB_11154540), anti-giantin (1:1000; #PA5-42884, RRID: AB_2607822), anti-WIPI1 (1:2000; #PA5-34973, RRID: AB_2552322), anti-GCP50 (1:1000; #MA5-25999, RRID: AB_2723827), anti-GRASP65 (1:5000; #PA3910, RRID: AB_2113207) (Thermo Scientific, Saint Louis, MO, USA);
- ER stress pathway: anti-p-38 MAPK α (1:1000; #PA5-37536, RRID: AB_2554145), anti-TRAF2 (1:1000;#PA5-20193, RRID: AB_11152352), anti-GADD34(1:1000; #PA1139, RRID: AB_2539894), anti-p-IRE1α (1:1000; #PA1-16927, RRID: AB_2262241), anti-p-PERK (1:1000; #PA5-40294, RRID: AB_2576881), anti-ATF6 (1:1000; #PA5-68556, RRID: AB_2688633), anti-p-ATF4 (1:1000; #PA5-36624, RRID: AB_2553621), anti-p-ASK1 (1:1000; #PA5-36619, RRID: AB_2553618), anti-p-CHOP (1:1000; #PA5-36796,RRID: AB_2553739), anti-p-EIF2α (1:1000; #MA5-15133; RRID: AB_10983400) (Thermo Scientific Saint Louis, MO, USA).
  Secondary antibodies: anti-rabbit (1:40,000; #A0545, RRID: AB_257896), or anti-mouse (1:40,000; #A9044, RRID: AB_258431) (Sigma -Aldrich, Saint Louis, MO, USA).

### Western blot analysis

Protein extraction and Western blot analysis were performed as described by Tabecka-Lonczynska et al. (2020) with some modification [23]. Briefly, 30 µg of testicular tissue homogenates were separated by using 10% SDS-PAGE electrophoresis. Next, the proteins were electroblotted onto methanol-activated polyvinylidene difluoride membranes (PVDF, Thermo Fisher Scientific, Poland) and blocked by using 1% BSA at room temperature for 1 h. Then, membranes were incubated overnight at 4 °C in the appropriate primary antibody and next for one hour in the secondary HRP-conjugated antibodies, presented above. The received blots were visualized using Western Blotting Luminol Reagent (Santa Cruz Biotechnology, Inc., Dallas, TX, USA) and LiCor C‑DiGit based on the attached instructions. GelQuantNET software was used for the densitometric analysis. The relative protein expression levels were quantified and normalized to β-actin (GelQuantNET software).

### qPCR analysis

RNA was extracted from testicular tissue using TRIzol reagent (Thermo Fisher Scientific, Waltham, MA, USA) following the manufacturer protocol. The quality of RNA was confirmed using a spectrophotometer (260/280 nm) and the integrity of obtained samples was controlled electrophoretically. Complementary DNA was synthesized from total RNA (1 μg) using High Capacity cDNA Reverse Transcription Kit (Thermo Fisher Scientific, Waltham, MA, USA) by following the attached instructions. For PCR amplification, 4 μl of autophagy-related primers (2 μl for forward and reverse primer) described in Table 1 (Genomed, Warsaw, Poland) was used. The PCR reaction was conducted for 35 cycles following conditions: 95 °C—10 min, 95 °C –35 s, annealing (specific temperature for each pair of primers) —35 s (Table 1), 72 °C – 35 s, 72 °C – 10 min. Obtained products were electrophoresed in 1.5% agarose gels and visualized using the Bio-Rad Gel Doc EZ Imager. The optical density was calculated with the use of GelQuantNET software and the results were normalized relatively to the expression of *ACTB* gene.

**Table 1:** Primers used for qPCR in this study

Target gene	Forward primer sequence (5’ – 3’)	Reverse primer sequence (5’ – 3’)	Annealing temp. (°C)
*mTOR*	ACCGGCACACATTTGAAGAAG	CACCACCAAGGATAAGGTAG	52.4
*ULK1*	AGGATGGGGACTTGGTTGC	CGATGTTTTCGTGCTTTAGTTCC	52.4
*PI3K*	CCTGGACATCAACGTGCAG	TGTCTCTTGGTATAGCCCAGAAA	53.2
*BECN1*	AGTTGAGAAAGGCGAGACAC	CACCACCAAGGATAAGGTAG	54.4
*BECN2*	GTCGCTACCGTCGTGACTTC	CAGACATGCACCTACCCAGC	55.9
*ATG16L*	CAGAGCAGCTACTAAGCGACT	AAAAGGGGAGATTCGGACAGA	52.4
*ATG13*	CAGAACTGCTGGTGAGGACACT	AGCAGGCTGATAGGAAAGGCGA	56,7
*ATG5*	AGCAACTCTGGATGGGATTG	CACTGCAGAGGTGTTTCCAA	55.3
*LC3*	CGAGAGCAGCATCCTACCAA	TTCTTCCGCGAATGTCGAGT	51.8
*ACTB*	CATCGGCAATGAGCGGTTCC	CCGTGTTGGCGTAGAGGTCC	68.1

### Statistical analysis

Presented results are reported as mean ± SD and statistical multiple comparisons were performed using GraphPad Prism ver. 6.0 (GraphPad Software Inc., California, USA). The data were assessed with one-way ANOVA followed by Dunnett's post hoc test in Western blot and qPCR analyses. A *p*-value of < 0.05 was considered statistically significant between groups treated with ALS and control or ALS with LPS and LPS control are presented as:*/^*p* < 0.05; **/^^*p* < 0.01; ***/^^^*p* < 0.001.

## Results

### Activation of ER stress response pathway in the mouse testis after ALS administration

**Fig. 1:**
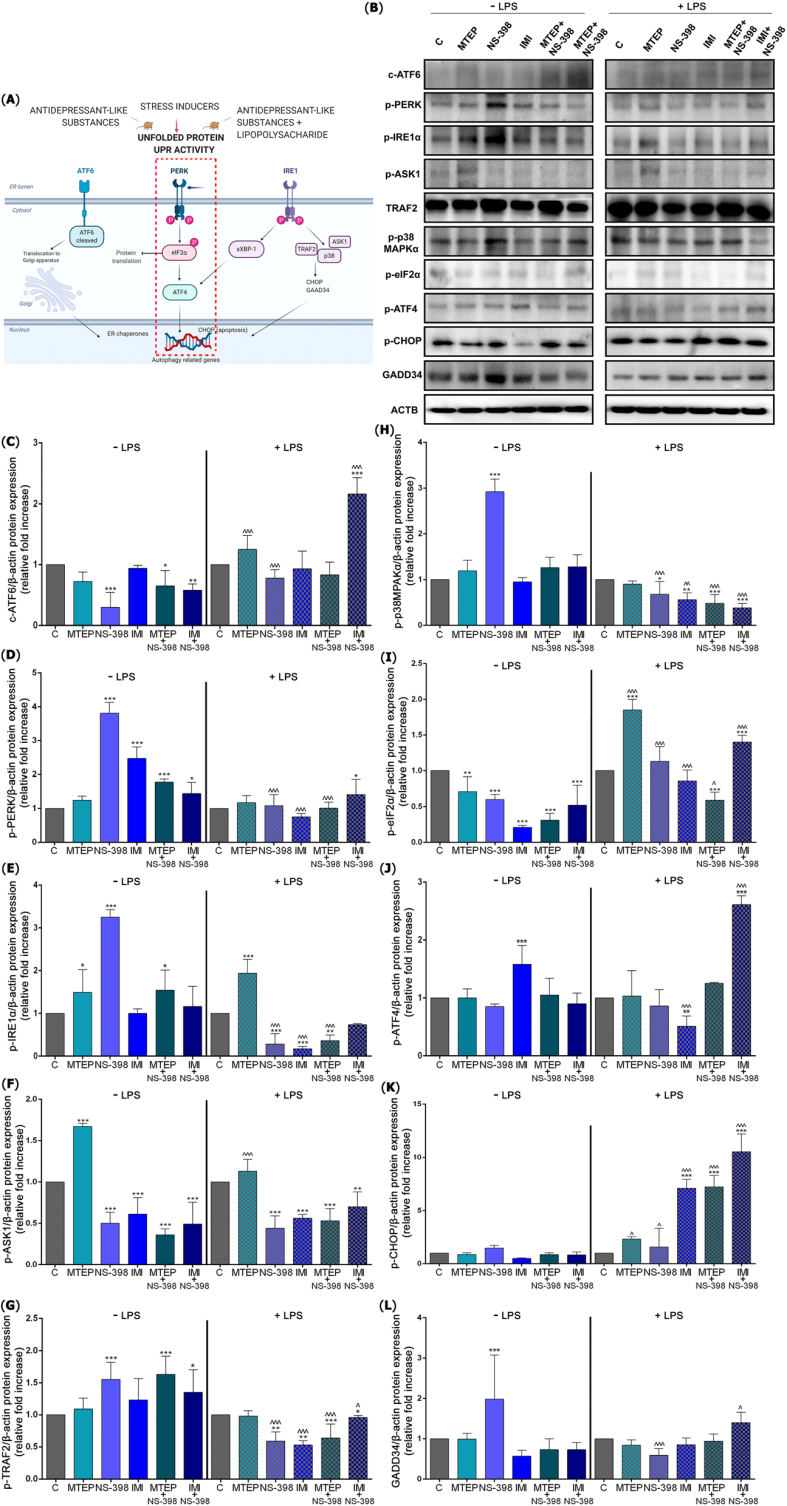
ALS- and LPS-induced endoplasmatic reticulum stress response in the mouse testis. (**A**) Schematic presentation of the ER-mediated stress response pathway in the testis after administration of ALS; expression of proteins involved in ATF6, PERK and IRE1 pathways examined by Western blot method (**B**); i.e.: ATF6 (**C**), p-PERK (**D**), p-IRE1α (**E**), p-ASK1 (**F**), p-TRAF2 (**G**), p-p38MAPKα (**H**), p-eIF2α (**I**), p-ATF4 (**J**), p-CHOP (**K**), GADD34 (**L**); Bars indicate mean $\pm$ SD, *n* = 7 per each group, (*) indicate comparison between control and analyzed groups after MTEP, NS-398, IMI, MTEP + NS-398, IMI + NS-398 with and without LPS; ***/^^^* p* < 0.001, **/^^* p* < 0.01, */^; (^) indicate comparison after administration the same substances between the groups without and with LPS; no indication = no statistical significance (one-way ANOVA with Dunnett’s a post hoc test). The results were normalized to β-actin

In this study, we observed downregulated level of c-ATF6 protein as a first of UPR arm, after NS-398, MTEP with NS-398, and IMI with NS-398 treatment (*p* < 0.001, *p* < 0.05 and *p* < 0.01, respectively), and the noted decreases were 3.33-fold, 1.53-fold and 1.72-fold (respectively) compared to control group. In contrast, we observed a significantly increased level of c-ATF6 synthesis after LPS treatment for the IMI with NS-398 (*p* < 0.001; 2.16-fold). Changes in c-ATF protein expression levels were observed between the same treatment without (w/o) and with (w/) LPS for MTEP (*p* < 0.001; 1.73-fold increase), NS-398 (*p* < 0.001; 2.6-fold increase), IMI with NS-398 (*p* < 0.001; 3.72-fold increase) [F(11,60) = 34.61] (Fig. 1C).

Upregulated phosphorylation of p-PERK was confirmed for NS-398 (*p* < 0.001; 3.81-fold), IMI (*p* < 0.001; 2.47-fold), MTEP with NS-398 (*p* < 0.001; 1.78-fold) and IMI with NS-398 (*p* < 0.05; 1.44-fold). The status of phosphorylation for PERK protein was elevated only after IMI with NS-398 (*p* < 0.05; 1.41-fold) treatment in LPS group. Decreases in p-PERK protein expression levels were observed between the same treatment w/o and w/ LPS for NS-398 (*p* < 0.001, 3.52-fold), IMI (*p* < 0.001; 3.25-fold), MTEP with NS-398 (*p* < 0.001; 1.76-fold) [F(11,60) = 69.61] (Fig. 1D).

The next sensor of UPR is IRE1α protein. The level of p-IRE1α was significantly increased after MTEP (*p* < 0.05;1.49-fold), NS-398 (*p* < 0.001; 3.25-fold) and MTEP with NS-398 combined treatment (*p* < 0.05; 1.54-fold). After LPS, active p-IRE1α was upregulated in MTEP group only (*p* < 0.001; 1.94-fold) and downregulated for NS-398 (*p* < 0.001; 5.88-fold), IMI (*p* < 0.001; 3.57) and MTEP with NS-398 (*p* < 0.01; 2.77-fold). Changes in p-IRE1α protein expression levels were observed between the same treatment w/o and w/ LPS for NS-398 (*p* < 0.001; 11.60-fold decrease), IMI (*p* < 0.001; 5.88-fold decrease) and MTEP with NS-398 (*p* < 0.01; 4.27-fold decrease) [F(11,60) = 53.61] (Fig. 1E).

Next, we observed increase in expression of p-ASK1 after MTEP (*p* < 0.001; 1.67-fold) and decrease for NS-398, IMI, MTEP with NS-398 and IMI with NS-398 (*p* < 0.001 for each; 2.0-fold, 1.63-fold, 2.77-fold, 2.04-fold respectively) compared to control. The decreased level was confirmed also in LPS group after NS-398 (*p* < 0.001; 2.27-fold), IMI (*p* < 0.001; 1.78-fold), MTEP with NS-398 (*p* < 0.001; 1.88-fold) and IMI with NS-398 (*p* < 0.01; 1.42-fold) compared to LPS control. Downregulated level of protein expression was observed only between MTEP and MTEP with LPS (*p* < 0.001; 1.47-fold) [F(11, 60) = 44.71] (Fig. 1F).

The level of p-TRAF2 expression was elevated, after NS-398 (*p* < 0.001; 1.55-fold), but also for combined therapy MTEP with NS-398, (1.63-fold increase) (*p* < 0.001) and IMI with NS-398 (*p* < 0.05; 1.35-fold increase). After LPS treatment we observed a significant decrease of p-TRAF2 expression in NS-398 (*p* < 0.01; 1.69-fold), IMI (*p* < 0.01; 1.88-fold), MTEP with NS-398 (*p* < 0.001; 1.56-fold) and IMI with NS-398 (*p* < 0.05; 1.04-fold) treated groups compared to LPS control. Decreases in protein expression levels were observed between the same treatment w/o and w/ LPS for NS-398 (*p* < 0.001; 2.62-fold), IMI (*p* < 0.001; 2.32-fold), MTEP with NS-398 (*p* < 0.001; 2.54-fold) and IMI with NS-398 (*p* < 0.05; 1.40-fold) [F(11,60) = 18.22] (Fig. 1G).

Next, we observed upregulated expression of p-p38MPAK in NS-398 group by 2.92-fold (*p* < 0.001) when compared to the control. Downregulated level of p-p38MPAK after LPS administration was observed for NS-398 (*p* < 0.05; 1.47-fold), IMI (*p* < 0.01; 1.78-fold), MTEP with NS-398 (*p* < 0.001; 2.08-fold) and in IMI with NS-398 (*p* < 0.001; 2.63-fold) group when compared to control LPS group. Changes in p-p38MPAK protein expression levels were observed after the same treatment w/o and w/ LPS in NS-398 (*p* < 0.001; 4.29-fold decrease), IMI (*p* < 0.01; 1.69-fold decrease), MTEP with NS-398 (*p* < 0.001; 2.625-fold decrease) and IMI with NS-398 (*p* < 0.001; 3.36-fold decrease) [F(11,60) = 76.69] (Fig. 1H).

Significant decrease in p-eIF2α protein expression was observed after administration of all substances in all study groups versus control consecutively MTEP (*p* < 0.01; 1.4-fold), NS-398 (*p* < 0.001; 1.66-fold), IMI (*p* < 0.001; 4.76-fold), MTEP with NS-398 (*p* < 0.001; 3.22-fold) and IMI with NS-398 (*p* < 0.001; 1.92-fold). The phosphorylated status of eIF2α was elevated after MTEP (*p* < 0.001; 1.85-fold) and IMI with NS-398 (*p* < 0.001; 1.4-fold) and reduced after MTEP with NS-398 (*p* < 0.001; 1.69-fold) versus LPS control. In p-eIF2α protein expression levels changes were observed between the same treatment w/o and w/ LPS for MTEP (*p* < 0.001; 2.60-fold increase), NS-398 (*p* < 0.001, 1.88-fold increase), IMI (*p* < 0.001; 4.09-fold increase), MTEP with NS-398 (*p* < 0.05; 1.90-fold increase) and IMI with NS-398 (*p* < 0.001; 2.69-fold increase) [F(11,60) = 63.86] (Fig. 1I).

Our analyses have shown upregulated level of phosphorylated p-ATF4 protein after IMI (*p* < 0.001; 1.58-fold) compared to control but interestingly, after LPS we observed significant decrease in p-ATF4 expression (*p* < 0.01; 1.96-fold). Moreover, after IMI with NS-398 treatment, the increase was noted (*p* < 0.001; 2.61-fold). Changes in p-ATF4 expression levels were observed between the same treatment w/o and w/ LPS for IMI (*p* < 0.001; 3.09-fold decrease) and IMI with NS-398 (*p* < 0.001; 2.90-fold increase) [F(60,11) = 34.39] (Fig. 1J).

The level of phosphorylated CHOP protein did not change in any analysed group (*p* > 0.05).The level of p-CHOP protein in LPS group was significantly increased after administration of ALS (IMI *p* < 0.001; 7.09-fold; MTEP with NS-398 *p* < 0.001; 7.22-fold; IMI with NS-398 *p* < 0.001; 10.53-fold). Increases in p-CHOP expression was observed between the same treatment w/o and w/LPS for MTEP (*p* < 0.05; 2.65-fold), NS-398 (*p* < 0.05; 1.06-fold), IMI (*p* < 0.001; 14.18-fold), MTEP with NS-398 (*p* < 0.001; 8.39-fold) and IMI with NS-398 (*p* < 0.001; 12.68-fold)[F(11,60) = 101.2] (Fig. 1K).

The expression of GADD34, another target protein, was importantly elevated only after NS-398 (*p* < 0.001; 7.12-fold increase) compared to control. No changes were found in the LPS group (*p* > 0.05). Changes in GADD34 protein expression levels were observed between the same treatment w/o and w/ LPS only for NS-398 (*p* < 0.001; 3.35-fold decrease) and IMI with NS-398 (*p* < 0.05; 1.91-fold increase) [F(11,60) = 7.154] (Fig. 1L). Scheme of the UPR signaling pathway in ER presented on Fig. 1A, and representative western immunoblots presented on Fig. 1B.

### Activation of Golgi apparatus stress response in the mouse testis after ALS administration

**Fig. 2:**
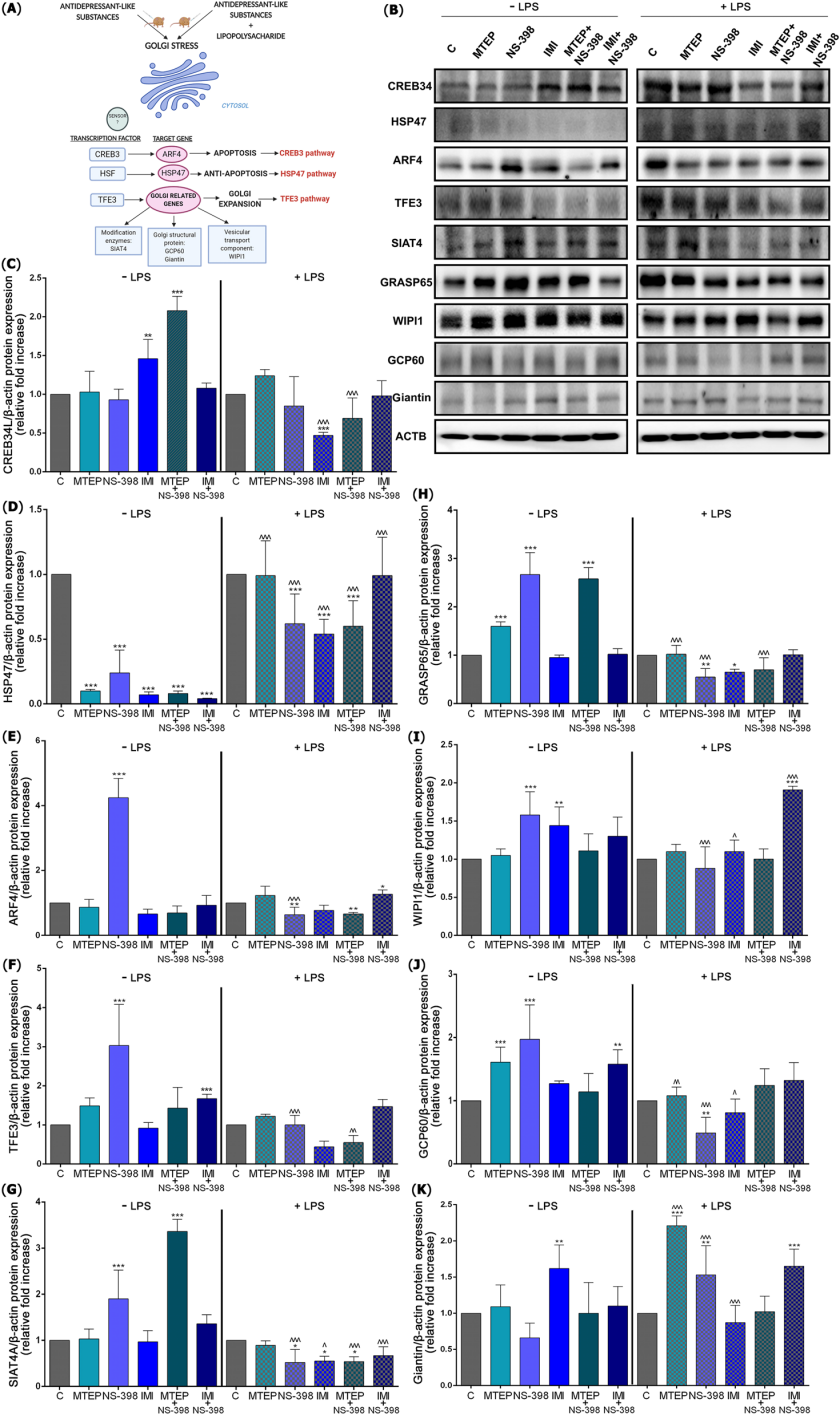
ALS- and LPS-induced Golgi apparatus stress response. (**A**) Schematic presentation of the Golgi-mediated stress response pathway in the testis after administration of ALS and LPS with ALS expression of proteins involved inCREB34, HSF47 and TFE3-mediated pathways evaluated with Western blot method (**B**), i.e.: CREB34L (**C**), ARF4 (**D**), HSP47 (**E**), TFE3 (**F**), SIAT4A (**G**), GRASP65 (**H**), WIPI1 (**I**), GCP60 (**J**), giantin (**K**). Bars indicate mean $\pm$ SD, *n* = 6 per each group; (*) indicate comparison between control and analyzed groups after MTEP, NS-398, IMI, MTEP + NS-398, IMI + NS-398 with and without LPS; (^) indicate comparison after administration the same substances between the groups without and with LPS; ***/^^^* p* < 0.001, **/^^* p* < 0.01, */^* p* < 0.05; no indication = no statistical significance (one-way ANOVA with Dunnett’s post hoc test). The results were normalized to β-actin

Next, we analyse the most important pathways of molecular regulatory mechanism of Golgi stress response mediated by TFE3, HSP47, and CREB3 [14] (Fig. 2A). At first, we controlled CREB34L pathway, but this protein was upregulated after IMI (*p* < 0.01; 1.46-fold) and MTEP with NS-398 (*p* < 0.001; 2.08-fold). However, under stress conditions, after LPS administration, the CREB34 pathway decreased only in IMI treatment group (*p* < 0.001; 2.12-fold). We also noted significant changes between the same treatment w/o and w/ LPS for CREB34L in the case of IMI (*p* < 0.001; 2.12-fold decrease) and MTEP with NS-398 (*p* < 0.001; 3.46-fold decrease) [F(11,60) = 26.19] (Fig. 2C).

The HSP47 protein was significantly reduced after MTEP (*p* < 0.001; 10.00-fold), NS-398 (*p* < 0.001; 4.16-fold), IMI (*p* < 0.001; 14.28-fold), MTEP with NS-398 (*p* < 0.001; 12.5-fold) and IMI with NS-398 (*p* < 0.001; 25.00-fold). Downregulated HSP47 level of protein expression was also observed after LPS and NS-398 (*p* < 0.001; 1.61-fold), IMI (*p* < 0.001; 1.85-fold) and MTEP with NS-398 (*p* < 0.001; 1.66-fold) versus LPS control. In contrast, increases in HSP47 expression levels were observed between the same treatment w/o and w/ LPS for MTEP (*p* < 0.001; 9.9-fold), NS-398 (*p* < 0.001; 2.58-fold), IMI (*p* < 0.001; 7.71-fold), MTEP with NS-398 (*p* < 0.001; 7.50-fold) and IMI with NS-398 (*p* < 0.001; 24.75-fold) [F(11,60) = 39.57] (Fig. 2D).

We observed upregulation of ARF4 protein after NS-398 (*p* < 0.001; 4.25-fold) relative to control. While, expression of ARF4 after LPS was significantly downregulated in NS-398 group (*p* < 0.01; 1.56-fold), MTEP with NS-398 (*p* < 0.01; 1.51-fold) and upregulated after IMI with NS-398 (*p* < 0.05; 1.25-fold) compared to control LPS group. After NS-398 with LPS the ARF4 expression was decreased related to NS-398 w/o LPS (*p* < 0.001; 6.64-fold) [F(11,60) = 96.03] (Fig. 2E).

The expression for TFE3 was significant elevated after NS-398 (*p* < 0.001; 3.3-fold increased) and after combined therapy for IMI with NS-398 (*p* < 0.001; 1.67-fold increase). No changes were noted after administration of LPS (*p* > 0.05). Decreases in TFE 3 expression levels were observed between the same treatment w/o and w/ LPS for NS-398 (*p* < 0.001; 3.03-fold) and MTEP with NS-398 (*p* < 0.01; 5.50-fold) [F(11,60) = 19.90] (Fig. 2F).

We also observed a significant increase in the expression level of post-translational modification enzymes. The upregulated level of SIAT4A was noted after NS-398 (*p* < 0.001; 1.9-fold increase) and MTEP with NS-398 (*p* < 0.001; 3.36-fold increase) compared to control. In contrast downregulated SIAT4A expression was observed after NS-398 (*p* < 0.05; 1.92-fold), IMI (*p* < 0.05; 1.81-fold) and MTEP with NS-398 (*p* < 0.05; 1.85-fold) versus LPS control group. Decreases in SIAT4A expression levels were observed between the same treatment w/o and w/ LPS for NS-398 (*p* < 0.001; 3.65-fold), IMI (*p* < 0.05; 1.76-fold), MTEP with NS-398 (*p* < 0.001; 6.22-fold) and IMI with NS-398 (*p* < 0.001; 2.02-fold) [F(11,60) = 61.32] (Fig. 2G).

Similarly, the level of GRASP65 was also upregulated after MTEP (*p* < 0.001; 1.6-fold), NS-398 (*p* < 0.001; 2.67-fold) and MTEP with NS-398 (*p* < 0.001; 2.58-fold) compared to control. While, expression of GRASP65 after LPS was reduced for NS-398 (*p* < 0.01; 1.81-fold) and IMI (*p* < 0.05; 1.53-fold) compared to LPS control. Decreases in GRASP65 expression levels were observed between the same treatment w/o and w/ LPS for MTEP (*p* < 0.001; 1.56-fold), NS-398 (*p* < 0.001; 4.85-fold) and MTEP with NS-398 (*p* < 0.001; 3.68-fold) [F(11,60) = 84.19] (Fig. 2H).

The WIPI1 expression showed a significant increase after NS-398 (*p* < 0.01; 1.58-fold) and IMI (*p* < 0.001; 1.44-fold) compared to control. Upregulated level was also observed after IMI with NS-398 related to LPS control (*p* < 0.001; 1.91-fold). Changes in WIPI1 expression levels were observed between the same treatment w/o and w/ LPS for NS-398 (*p* < 0.001; 1.79-fold decrease) IMI (*p* < 0.05; 1.30-fold decrease) and IMI with NS-398 (*p* < 0.001; 1.46-fold increase) [F(11,60) = 15.99] (Fig. 2I).

Structural protein of the Golgi apartment, GCP60, revealed the upregulated level after MTEP (*p* < 0.001; 1.61-fold), NS-398 (*p* < 0.001; 1.97-fold) and IMI with NS-398 (*p* < 0.01; 1.58-fold) compared to control. In contrast GCP60 expression showed downregulation relative to the LPS control only after NS-398 (*p* < 0.01; 2.04-fold). Decreases in GCP60 levels were observed between the same treatment w/o and w/ LPS for MTEP (*p* < 0.01; 1.49-fold), NS-398 (*p* < 0.001; 4.02-fold) and IMI (*p* < 0.05; 1.56-fold) [F(11,60) = 14.33] (Fig. 2 J).

For giantin expression profile, the second structural protein, the level increased after IMI (*p* < 0.01; 1.62-fold) compared to control. Similarly, upregulated level of giantin was presented after MTEP (*p* < 0.001; 2.21-fold), NS-398 (*p* < 0.01; 5.3-fold) and IMI with NS-398 (*p* < 0.001; 1.65-fold) compared to LPS control group. Changes in giantin expression levels were observed between the same treatment w/o and w/ LPS for MTEP (*p* < 0.001; 2.02-fold increase), NS-398 (*p* < 0.001; 2.31-fold increase) and IMI (*p* < 0.001; 1.86-fold decrease) [F(11,60) = 16.26] (Fig. 2 K). Western immunoblots are presented on Fig. 2 B.

### Activation of autophagy pathway in the mouse testis after ALS administration

**Fig. 3:**
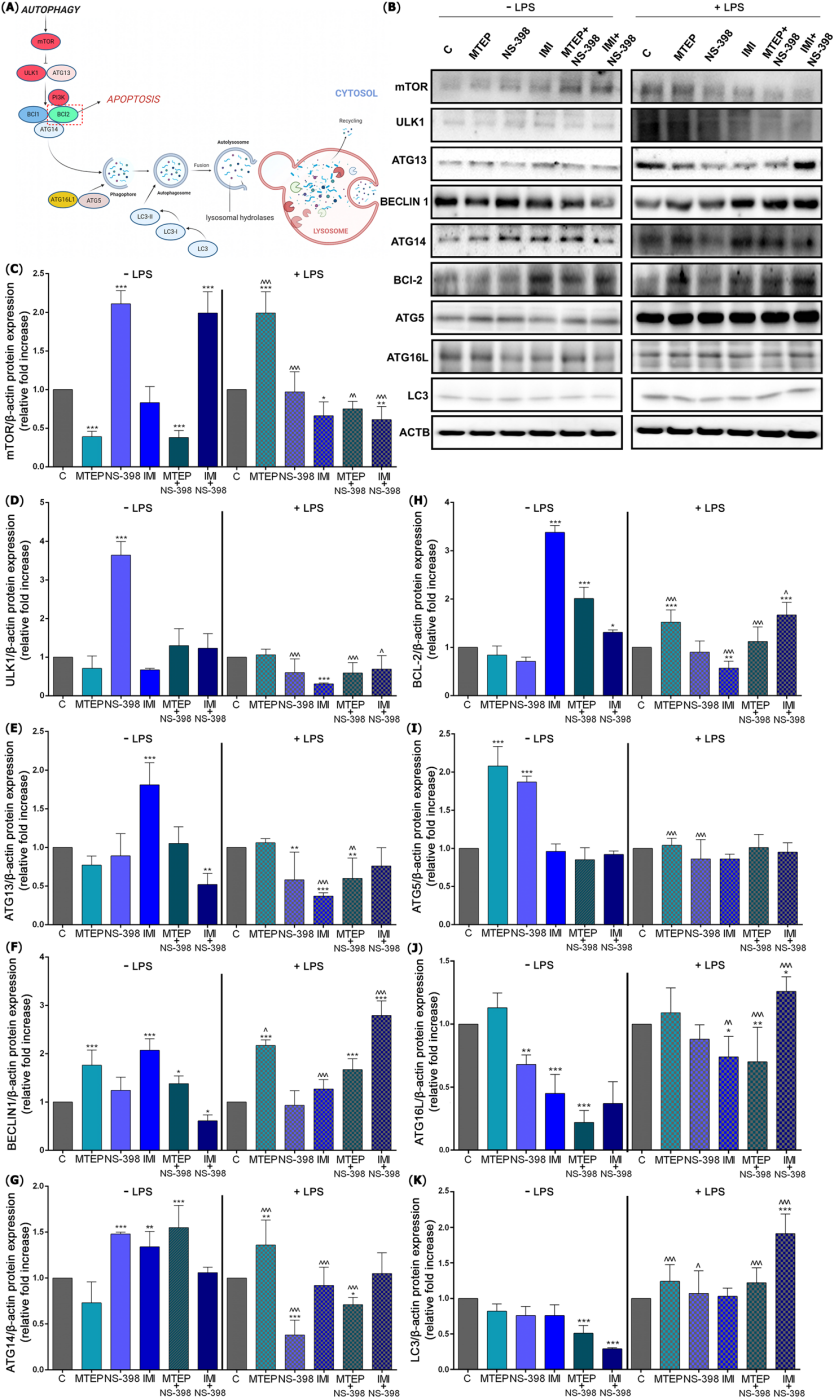
ALS- and LPS-induced activation of autophagy pathway. (**A**) Schematic presentation of the autophagy pathway in the testis after administration of ALS; expression of proteins involved in autophagy initiation evaluated with Western blot method (**B**), i.e.: mTOR (**C**), ULK1(**D**), ATG13 (**E**), BECLIN1 (**F**), ATG14 (**G**), BCl-2 (**H**), ATG5 (**I**), ATG16L (**J**), LC3 (**K**). Bars indicate mean $\pm$ SD, *n* = 7 per each group; (*) indicate comparison between control group and analyzed groups after MTEP, NS-398, IMI, MTEP + NS-398, IMI + NS-398 with and without LPS; (^) indicate comparison after administration the same substances between the groups without and with LPS; ***/^^^* p* < 0.001, **/^^* p* < 0.01, */^* p* < 0.05; no indication = no statistical significance (one-way ANOVA with Dunnett’s post hoc test). The results were normalized to β-actin

Next, we performed a comprehensive analysis of proteins expression of the autophagy metabolic pathway (Fig. 3A). In detail, after NS-398 treatment (*p* < 0.001; 2.11-fold) and IMI with NS-398 (*p* < 0.001; 1.99-fold) the mTOR expression was significantly higher than control. However, mTOR protein expression was reduced after MTEP (*p* < 0.001; 2.56-fold) and MTEP with NS-398 (*p* < 0.001; 2.63-fold). Also, MTEP (*p* < 0.001; 1.98-fold increase) therapy resulted in a significantly elevated level of mTOR versus control LPS group, but after IMI (*p* < 0.05; 1.51-fold) and IMI with NS-398 (*p* < 0.01; 1.63-fold) the protein expression was reduced. Changes in protein expression levels for mTOR were observed after the same treatment w/o and w/ LPS for MTEP (*p* < 0.001; 5.10-fold increase), NS-398 (*p* < 0.001; 2.17-fold decrease), MTEP with NS-398 (*p* < 0.01; 1.97-fold increase), IMI with NS-398 (*p* < 0.001; 3.26-fold decrease) [F(11.60) = 74.19] (Fig. 3C).

Further, only after NS-398 (*p* < 0.001; 3.64-fold) the level of ULK1 protein expression was increased. After LPS, the downregulated level of ULK1 revealed for IMI (*p* < 0.001; 3.22-fold). Decreases for ULK1 were observed after the same treatment w/o and w/ LPS for NS-398 (*p* < 0.001; 6.06-fold), MTEP with NS-398 (*p* < 0.001; 2.20-fold) and IMI with NS-398 (*p* < 0.05; 1.78-fold) [F(11,60) = 58.87] (Fig. 3D).

However, an increased level of ATG13 after IMI (*p* < 0.001; 1.81-fold) and reduced after IMI with NS-398 (*p* < 0.01; 1.92-fold) was found compared to control. The levels of ATG13 protein after NS-398 (*p* < 0.01; 1.72), IMI (*p* < 0.001; 2.7-fold) and MTEP with NS-398 (*p* < 0.01; 1.66-fold) were downregulated versus control LPS group. Decreases in ATG13 expression was observed after the same treatment w/o and w/LPS for IMI (*p* < 0.001; 4.89-fold) and MTEP with NS-398 (*p* < 0.01; 1.75-fold) [F(11.60) = 20.06] (Fig. 3E).

After MTEP, IMI (p < 0.001; 1.76-fold and 2.07-fold respectively) and MTEP with NS-398 (*p* < *p* < 0.05; 1.38-fold) a significant increase in BECLIN1 expression was observed compared to control, while decrease was observed only after IMI with NS-398 (*p* < 0.05; 1.63-fold). For BECLIN1 we observed a significantly elevated level after MTEP, MTEP with NS-398 and IMI with NS-398 (*p* < 0.001 for each; 2.17-fold, 1.67-fold, 2.79-fold, respectively) compared to LPS control group. Changes in BECLIN1 expression was observed after the same treatment with LPS for MTEP (*p* < 0.05; 1.23-fold increase), IMI (*p* < 0.001; 1.62-fold decrease) and IMI with NS-398 (*p* < 0.001; 4.57-fold increase) [F(11,60) = 50.52] (Fig. 3F).

In contrast, the level of ATG14 was significantly upregulated after NS-398 (*p* < 0.001; 1.48-fold), IMI (*p* < 0.01; 1.34-fold) and MTEP with NS-398 (*p* < 0.001; 1.55-fold) versus control. High ATG14 expression relative to the LPS control group was shown only after MTEP (*p* < 0.01) and this level was 1.36-fold higher. The reduced level of ATG14 was observed after NS-398 (*p* < 0.001; 2.63-fold) and MTEP with NS-398 (*p* < 0.05; 1.4-fold) compared to LPS control. Changes in protein expression levels for ATG14 were observed after the same treatment w/o and w/ LPS for MTEP (p < 0.001; 1.86-fold increase), NS-398 (*p* < 0.001; 3.89-fold decrease), IMI (*p* < 0.001; 1.45-fold decrease) and MTEP with NS-398 (*p* < 0.001; 2.18-fold decrease) [F(11,60) = 25.32] (Fig. 3G).

Subsequently, upregulated BCl-2 protein expression was observed after IMI, MTEP with NS-398 (*p* < 0.001 for both; 3.38-fold, 2.01-fold respectively) and IMI with NS-398 (*p* < 0.05; 1.31-fold) versus control. After LPS, elevated BCl-2 level was found after MTEP (*p* < 0.001; 1.52-fold), and IMI with NS-398 administration (*p* < 0.001; 1.67-fold) and downregulated after IMI (*p* < 0.01; 1.75-fold) compared to LPS control. Changes in protein expression levels for BCl-2 were observed after the same treatment w/o and w/ LPS for MTEP (*p* < 0.001; 1.80-fold increase), IMI (*p* < 0.001; 5.92-fold decrease), MTEP with NS-398 (*p* < 0.001; 1.79-fold decrease) and IMI with NS-398 (*p* < 0.05; 1.27-fold increase) [F(11,60) = 101.7] (Fig. 3H).

Next, the level of ATG5, was elevated after administration of MTEP (*p* < 0.001; 2.08-fold increase), and NS-398 compared to control (*p* < 0.001; 1.87-fold increase). The level of ATG5 expression did not change in any group analyzed after ALS administration in LPS groups (*p* > 0.05). Decrease in ATG5 expression was observed after the same treatment w/o and w/ LPS for MTEP (*p* < 0.001; 2.00-fold), and for NS-398 (*p* < 0.001; 2.17-fold) [F(11,60) = 52.41] (Fig. 3I).

The ATG16L protein level, associated with expanding of vesicle membrane was markedly reduced. The levels after NS-398 (*p* < 0.01; 1.47-fold), IMI (*p* < 0.001; 2.22-fold) and MTEP with NS-398 (*p* < 0.001; 4.54-fold) were significantly lower than control. While in stress conditions after IMI with NS-398, the level of ATG16L increased significantly and was 1.26-fold higher than LPS control group (*p* < 0.05). After IMI (*p* < 0.05; 1.35-fold) and MTEP with NS-398 (*p* < 0.01; 1.42-fold) administration, ATG16L protein expression was decreased compared to LPS control group. Changes in ATG16L levels were observed after the same treatment w/o and w/LPS for IMI (*p* < 0.01; 1.64-fold increase), MTEP with NS-398 (*p* < 0.001; 3.18-fold increase) and IMI with NS-398 (*p* < 0.001; 2.00-fold increase) [F(11,60) = 30.65] (Fig. 3J).

We observed reduced expression of LC3 protein after MTEP with NS-398 treatment (*p* < 0.001; 1.96-fold) and IMI with NS-398 treatment (*p* < 0.001; 3.44-fold decrease) versus control group. Whereas, the LC3 protein expression increased only after IMI with NS-398 (*p* < 0.001; 1.91-fold) compared to LPS control group. Increases in protein expression levels for LC3 were observed after the same treatment w/o and w/ LPS for MTEP (*p* < 0.001; 1.51-fold), NS-398 (*p* < 0.05; 1.40-fold), MTEP with NS-398 (*p* < 0.001; 2.39-fold) and IMI with NS-398 (*p* < 0.001; 6.58-fold) [F(11,60) = 33.96] (Fig. 3K).

**Fig. 4:**
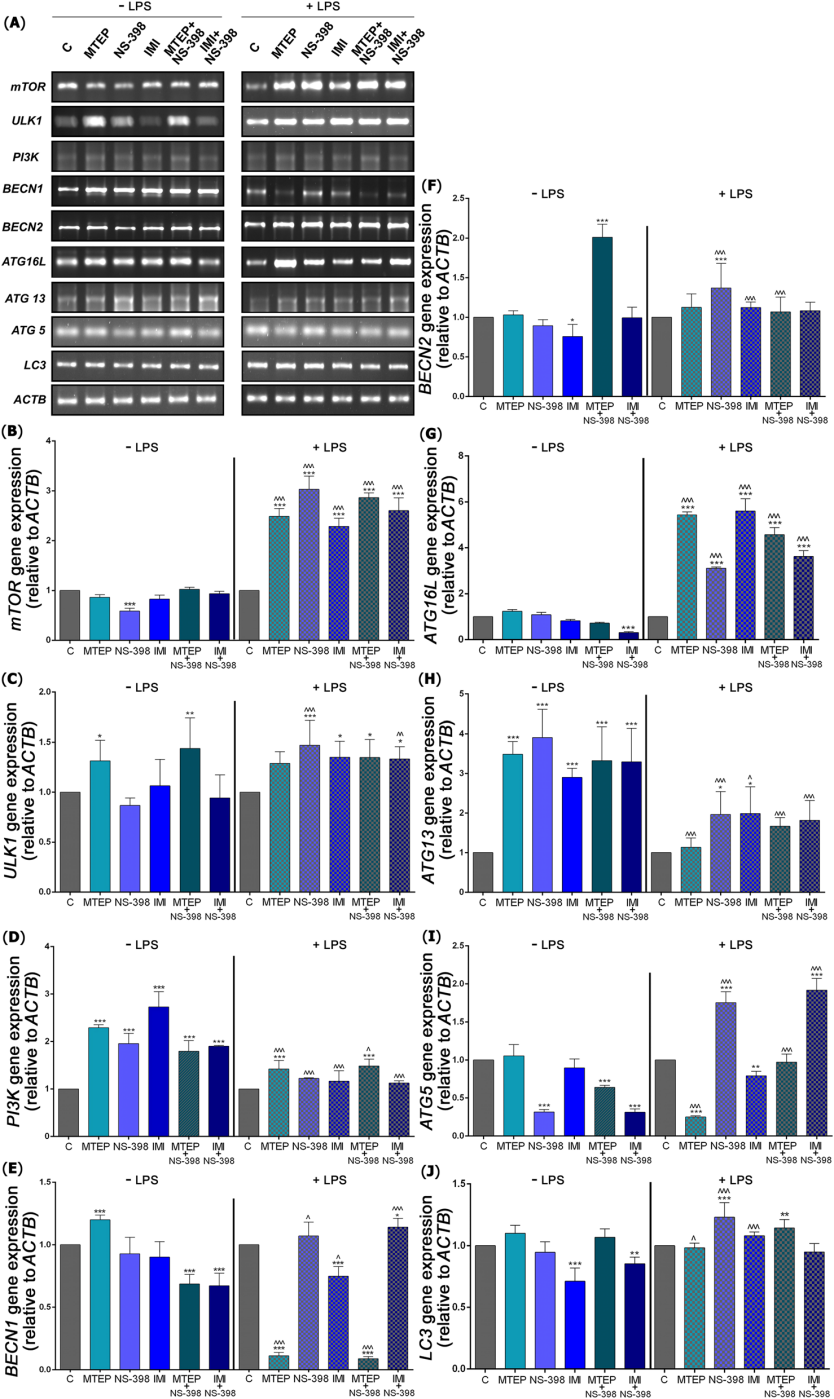
mRNA levels encoding autophagy pathway initiation proteins in mice testis after ALS administration. (**A**) expression of genes involved in autophagy initiation evaluated with qPCR method, i.e.: *mTOR (***B**), *ULK1 *(**C**), *PI3K* (**D**), *BECN1 *(**E**), *BECN2 *(**F**), *ATG16L* (**G**), *ATG13* (**H**), *ATG5 *(**I**), *LC3* (**J**). Bars indicate mean $\pm$ SD, *n* = 7 per each group; (*) indicate comparison between control group and analyzed groups after MTEP, NS-398, IMI, MTEP + NS-398, IMI + NS-398 with and without LPS, and comparison between the same substance in analyzed group; (^) indicate comparison after administration the same substances between the groups without and with LPS; ***/^^^* p* < 0.001, **/^^* p* < 0.01, */^* p* < 0.05; no indication = no statistical significance (one-way ANOVA with Dunnett’s post hoc test). The results were normalized to *ACTB*

Simultaneously, qPCR analyzes were carried out to compare the levels of expression for genes related to autophagy (Fig. 4A). For *mTOR* gene expression the level was reduced only after NS-398 administration (*p* < 0.001; 1.70-fold), compared to control, however, it increased significantly in all LPS groups: MTEP (*p* < 0.001; 2.48-fold), NS-398 (*p* < 0.001; 3.03-fold), IMI (*p* < 0.001; 2.28-fold), MTEP with NS-398 (*p* < 0.001; 2.86-fold) and IMI with NS-398 (*p* < 0.001; 2.60-fold) related to LPS control. Increases in *mTOR* gene expression levels were observed after the same treatment w/o and w/LPS for MTEP (*p* < 0.001; 2.88-fold), NS-398 (*p* < 0.001; 5.22-fold), IMI (*p* < 0.001; 2.78-fold), MTEP with NS-398 (*p* < 0.001; 2.80-fold) and IMI with NS-398 (*p* < 0.001; 2.79-fold) [F(11,60) = 292.3] (Fig. 4B).

Increased *ULK1* gene expression was noted after MTEP (*p* < 0.05; 1.31-fold) and MTEP with NS-398 (*p* < 0.01; 1,43-fold) compared to control and in stress conditions elevated after NS-398 (*p* < 0.001; 1.47-fold), IMI (*p* < 0.05; 1.34-fold), MTEP with NS-398 (*p* < 0.05; 1.34-fold) and IMI with NS-398 (*p* < 0.05; 1.33-fold) versus control LPS group. Increased level of *ULK1* expression was observed after the same treatment w/o and w/LPS for NS-398 (*p* < 0.001; 1.70-fold) and IMI with NS-398 (*p* < 0.001; 2.79-fold) [F(11,60) = 7.77] (Fig. 4C).

For *PI3K* gene, the study demonstrated an increase in expression after all ALS, in detail MTEP (2.29-fold), NS-398 (1.95-fold), IMI (1.72-fold), MTEP with NS-398 (1.79-fold) and IMI with NS-398 (1.90-fold) (*p* < 0.001 for each) related to control. After LPS, the increase was observed after MTEP (*p* < 0.001; 1.42-fold) and MTEP with NS-398 (*p* < 0.001; 1.48-fold) compared to LPS control. Decreased level of *PI3K* gene expression was observed after the same treatment w/o and w/LPS for MTEP (*p* < 0.001; 1.61-fold), NS-398 (*p* < 0.001; 1.59-fold), IMI (*p* < 0.001; 2.34-fold), MTEP with NS-398 (*p* < 0.05; 1.20-fold) and IMI with NS-398 (*p* < 0.001; 1.69-fold) [F(11,60) = 69.79] (Fig. 4D).

For *BECN1* gene, increased expression was confirmed after MTEP (*p* < 0.001; 1.19-fold) and reduced after MTEP with NS-398 (*p* < 0.001; 1.47-fold) and IMI with NS-398 (*p* < 0.001; 1.49-fold) compared to control. However, after LPS the expression was reduced after MTEP (*p* < 0.001; 9.09-fold), IMI (*p* < 0.001; 1.33-fold) and MTEP with NS-398 (*p* < 0.001; 12.5-fold) and upregulated after IMI with NS-398 (*p* < 0.05; 1.14-fold) administration compared to LPS control. Changes in *BECN1* gene expression level were observed after the same treatment w/o and w/ LPS for MTEP (*p* < 0.001; 10.81-fold decrease), NS-398 (*p* < 0.05; 1.16-fold increase), IMI (*p* < 0.001; 1,21-fold decrease), MTEP with NS-398 (*p* < 0.001; 7.72-fold decrease) and IMI with NS-398 (*p* < 0.001; 1.70-fold increase) [F(11,60) = 129.7] (Fig. 4E).

The downregulated expression of the *BECN2* gene was observed after IMI (*p* > 0.05; 1.33-fold) and upregulated after MTEP with NS-398 (*p* < 0.001; 2.01-fold) compared to control. After LPS, the *BECN2* gene expression increase was observed after NS-398 administration only (*p* < 0.001; 1.36-fold). Changes in *BECN2* gene expression level was observed after the same treatment w/o and w/ LPS for NS-398 (*p* < 0.001; 1.52-fold increase) and IMI (*p* < 0.001; 1.49-fold increase) and MTEP with NS-398 (*p* < 0.001; 1.89-fold decrease) [F(11,60) = 28.27] (Fig. 4F).

For *ATG16L,* the expression decreased after IMI with NS-398 combined therapy(*p* < 0.001; 3.22-fold) compared to control. However, after LPS the expression was increased after MTEP (5.43-fold), NS-398 (3.11-fold), IMI (5.60-fold), MTEP with NS-398 (4.57-fold) and IMI with NS-398 (3.62-fold) (*p* < 0.001 for each). Increase in *ATG16L* gene expression level was observed after the same treatment w/o and w/ LPS for MTEP (*p* < 0.001; 4.41-fold), NS-398 (*p* < 0.001; 2.87-fold), IMI (*p* < 0.001; 6.82-fold), MTEP with NS-398 (*p* < 0.001; 6.43-fold) and IMI with NS-398 (*p* < 0.001; 11.67-fold) [F(11,60) = 570.6] (Fig. 4G).

*ATG13* gene expression level was significantly higher for all groups after ALS, in detail MTEP (3.48-fold), NS-398 (3.90-fold), IMI (2.89-fold), MTEP with NS-398 (3.32-fold), IMI with NS-398 (3.29-fold) (*p* < 0.001 for each) compared to control and after LPS therapy significantly higher for NS-398 (*p* < 0.05; 1.96-fold) and IMI (*p* < 0.05; 1.98-fold) compared to LPS control (Fig. 4H). In addition, decreased level of *ATG13* gene expression was observed after the same treatment w/o and w/LPS for MTEP (*p* < 0.001; 3.07-fold), NS-398 (*p* < 0.001; 1.98-fold), IMI (*p* < 0.05; 1.45-fold), MTEP with NS-398 (*p* < 0.001; 2.00-fold) and IMI with NS-398 (*p* < 0.001; 1.81-fold)[F(11,60) = 24.06] (Fig. 4H).

The level of *ATG5* expression was significantly lower in NS-398 (*p* < 0.001; 3.22-fold), MTEP with NS-398 (*p* < 0.001; 1.58-fold) and IMI with NS-398 (*p* < 0.001; 3.21-fold) related to control group and after LPS in MTEP (*p* < 0.001; 4.16-fold) and IMI (*p* < 0.01; 1.26-fold) groups, but in NS-398 (*p* < 0.001; 1.75-fold) and IMI with NS-398 (*p* < 0.001; 1.91-fold) was increased related to LPS control. Changes in *ATG5* were observed after the same treatment w/o and w/LPS for MTEP (*p* < 0.001; 4.37-fold decrease), NS-398 (*p* < 0.001; 5.64-fold increase), IMI with NS-398 (*p* < 0.001; 1.52-fold increase) and IMI with MTEP (*p* < 0.001; 6.16-fold increase) [F(11,60) = 196.5] (Fig. 4I).

Finally, decrease in *LC3* expression was found after IMI (*p* < 0.001; 1.40-fold) and IMI with NS-398 (*p* < 0.01; 1.17-fold) compared to control. After LPS, the increase was observed for NS-398 (*p* < 0.001; 1.23-fold) and MTEP with NS-398 (*p* < 0.01; 1.14-fold) compared to LPS control. Changes in *LC3* were noted after the same treatment w/o and w/ LPS for MTEP (*p* < 0.05; 1.12-fold decrease), NS-398 (*p* < 0.001; 1.30-fold increase) and IMI (*p* < 0.001; 1.52-fold increase) [F(11,60) = 24.06] (Fig. 4J).

## Discussion

Our previous investigation into the impact of antidepressants on testicular growth factors revealed a negative effect [24]. Given the crucial role of autophagy in cell survival and fate determination, we aimed to elucidate the intricate metabolic pathways involving the ER, Golgi apparatus, and autophagy in response to ALS and ALS combined with LPS. Our study presents novel findings regarding the effect of different substances on the activation of ER and Golgi apparatus stress responses, indicating that the mechanism is substance-specific. Specifically, we observed the activation of the IRE1 pathway branch in response to NS-398 and MTEP with NS-398 treatment, while a reduction in p-IRE1 activity was noted for both substances administered with LPS. In contrast, phosphorylation of PERK was noted following almost all ALS treatments, but no effect on the PERK-CHOP axis activation was observed. Furthermore, our results indicate that testicular cells are directed towards the apoptotic pathway after IMI with NS-398 and LPS, as evidenced by the upregulation of phosphorylated CHOP and ATF4 protein levels. It is known that the UPR may lead to mitochondrial-dependent or -independent apoptosis [25, 26], and in our studies, an increase in the active form of CHOP was observed after IMI with NS-398 and LPS, which is likely inhibited by the high level of BCl-2 protein observed [27]. Interestingly, our data suggest that IMI with NS-398 and LPS directs the analyzed testicular cells towards the autophagy pathway.

Wang et al. (2019) have demonstrated that the level of cyclic AMP-responsive element-binding protein 3 (CREB3) in Leydig cells increases with age. Conversely, a decrease in CREB3 levels results in elevated expression of genes responsible for steroidogenesis and testosterone synthesis. Inhibition of CREB3 expression affects steroidogenic enzymes and genes encoding cell cycle factors, indicating a crucial role of CREB3 in the regulation of reproduction [5, 28]. Moreover, oxidative stress induces ER stress, leading to the activation of the UPR [29]. Initially, UPR is a protective response; however, when activated for an extended period, it leads to activation of various signaling pathways such as IRE1, PERK, ATF6, and CHOP, which subsequently determine the fate of the cell [30]. So far it is known, that certain antidepressants or imipramine can increase antioxidant enzyme activity and reduce lipid peroxidation, a marker of oxidative stress [31, 32]. It also was shown, that LPS administration increased oxidative stress markers, disrupt redox balance, and promote tissue injury in various organs [33–35]. However, the precise role and its interplay with antidepressant treatment or LPS-induced inflammation are still areas of active research and unfortunately not much mechanistic data is available.

Other studies have also reported on the effects of pharmacological substances on ER stress via different mechanisms. Disruption of ER function leads to disturbance of calcium homeostasis, resulting in abnormal protein folding, ER stress, and UPR activation [36]. Cisplatin, a drug-induced toxicity treatment, induces ROS-mediated ER stress in the testis [6, 37]. Similarly, oxidative stress induced after cisplatin administration in the testis activates the ER stress and MAPK pathway [38]. In vitro studies on Leydig cells in mice revealed stress-mediated apoptosis and autophagy through the activation of ER stress proteins PERK, eIF-2α, and ATF4, apoptosis-relative protein caspase-3, caspase-7, caspase-12, LC3II, and ATG5 [39]. The effects of adriamycin also revealed testicular dysfunction through crosstalk between ER stress and the mitochondrial signaling pathway [40]. Busulfan, another tested drug, caused apoptosis in testicular germ cells in mice and the C18-4 cell line (mouse spermatogonial stem cell line) by inducing the IRE1 and PERK pathways [41]. Also, finasterides, a 5α-reductase inhibitor, was reported to activate apoptosis in germ cells by ROS-mediated ER stress [42]. Fluoxetine has been reported as an inducer of ER stress-underlying autophagy in cancer cells while, also serving as an antidepressant therapy [43].The results of these studies suggest that the proper functioning and regulation of reproductive cells may depend on the substance used and its effects on ER stress and the UPR pathway.

The p38MAPK is activated by oxygen species-sensitive ASK1 signaling complex [44], which is reflected in our results mainly after LPS treatment. Further, ULK1 is ready to act by p38MAPK by blocking the attachment to the ATG13 effector leads to inhibition of autophagy [45]. Our results revealed an upregulation of PERK-ATF4-CHOP axis factors after treatment with IMI and NS-398, and LPS only, which confirms the transcriptional regulation of autophagy genes. Others also supported our findings [46]. Specifically, our study found that the CHOP protein level was significantly upregulated only after IMI and NS-398 combined therapy compared to the control group. However, prolonged CHOP activation can lead to apoptosis and limit autophagy [47]. Other studies have shown a close relationship between CHOP overactivation and Golgi fragmentation [48, 49], which is a consequence of ER stress and the direction of numerous secretory proteins to the Golgi apparatus. This excess of proteins triggers Golgi complex stress response, which was presented in detail by Taniguchi et al. [48].

In our study, we also observed the activation of TFE3, HSP47, and CREB34 signaling pathways in response to various stress factors. Specifically, we confirmed the activation of the CREB34 and TFE3 pathways after ALS treatment. The CREB34 axis path branch was upregulated only after IMI and NS-398, whereas TFE3 was elevated after NS and IMI with NS-398. However, no activation of either pathway was observed after ALS administration in the LPS group. Subsequently, the initiation of the ULK1/ATG13/FIP200 complex triggers the autophagy pathway, which leads to the formation and degradation of autophagosomes [50]. Our study found that the ULK1 protein was upregulated only after NS-398 compared to the control group, while ATG13 activity was decreased via two known mechanisms related to phosphorylation of ATG13 on Ser-258 by mTOR and Ser-224 by MAPK pathway [51]. After administration of NS-398, IMI and MTEP with NS-398 in LPS group, the ATG13 was clearly reduced. This data may indicate that in ATG13 deficient cells under stress conditions, the elevated level of caspase activity is necessary for the initiation of successful apoptotic cell death [52, 53]. However, our research showed an increase in the level of BECLIN1, ATG16L, and LC3 proteins, which are important markers of autophagosome formation [23] after IMI with NS-398 and LPS treatment. The observed outcomes of this particular combination may in part suggest a favorable impact on the functionality of testicular cells.

Several experiments have been carried out on male testes to investigate the process of autophagy. It has been shown by Yi et al. (1995) that autophagy occurs more frequently in Leydig cells, which are involved in steroid synthesis, compared to other cell types [54]. Autophagy disturbance in these cells impairs testosterone synthesis by affecting lipid metabolism regulation and the reuptake of cholesterol [14]. Moreover, decrease in the activity of the autophagy process by knockout of ATG7 and ATG5 in steroidogenic cells leads to a disturbance of sexual behavior in older male mice, which was also confirmed by a significant decrease in serum testosterone levels. The level of autophagy does not appear to be influenced by Leydig cell differentiation at different developmental stages [14].

The process of autophagy in testicular cells was also observed after the administration of various pharmacological substances. Foldman et al. (1980) found that pentobarbital administration in rats decreased membrane levels and increased autophagic bodies even after discontinuation of the drug [55]. Similarly, Yang et al. (2017) observed decreased spermatogenesis activity and androgen levels in Leydig cell cultures in vitro after rapamycin treatment [56]. The above information and the decrease in the activity of the autophagy pathway observed in our research seem to explain the dysfunction of the male reproductive system (decrease in sperm quality and fertility) after the use of the most analyzed antidepressants. Activation of the autophagy pathway in response to IMI with NS-398 and LPS treatment suggests that this combined therapy may promote normal physiological processes in the testis during depression therapy. This was also confirmed by our results of gene expression for *BECN1* and *ATG16L* after IMI with NS-398 and LPS. It's quite clear that male fertility is related to the quality of the sperm produced in the testicles. Mature sperm does not have an ER, however, in the process of their maturation, ER stress can be triggered and may play an important role in fertilization. The testis-specific bZip type transcription factor, Tisp40, is upregulated during spermatogenesis in rat testis [57, 58], and is responsible for regulating sperm maturation through the UPR system survival mechanisms against stress-induced apoptosis. Other genes, such as *RAD51*, *TOP1*, and *TOP2A*, are also involved in regulating metabolic pathways under cellular stress, and their expression levels may indicate different susceptibilities of germ cells to DNA-damaging compounds [59–62].

In the present study, we noted the clear effect of pharmacological substances with antidepressant potential alone and with LPS on the course of metabolic processes in the ER, Golgi apparatus, and lysosomes of mouse testicular cells. Our findings offer a comprehensive understanding of ER and Golgi stress and autophagy dynamics following ALS treatment in a mouse model of depression. The observed data suggests that the concomitant administration of IMI and NS-398 as part of an antidepressant therapy may not impede reproductive capacity through the appropriate modulation of reproductive processes via autophagy induction. Hence, we can be postulated that the upregulation of protein expression within the examined cellular pathways is influenced by multiple factors, and currently, there is no identified singular decisive mechanism that governs the destiny of male reproductive cells. We anticipate that our results will provide insights into the role of ER and Golgi apparatus stress in male infertility associated with antidepressant therapy.

### Supplementary Information

**Additional file 1.**

## Data Availability

Data will be made available on request.

## Abbreviations

ALS: Antidepressant-like substances
AMPK: 5’-AMP-activated protein kinase
ASK1: Apoptosis signal-regulating kinase 1
ATF: Activating transcription factor
ARF4: Adp-Ribosylation Factor 4
ATG: Autophagy-related protein
BCl-2: B-cell lymphoma 2
C18-4 cell line: Mouse spermatogonial stem cell line
CHOP: C/EBP homologous protein
CREB34: Cyclic AMP-responsive element-binding protein 34
DMSO: Dimethyl sulfoxide
eIF2α: Eukaryotic initiation factor
ER: Endoplasmic reticulum FIP200, focal adhesion kinase family interacting protein of 200 kD
HSP47: Heat shock protein 47
IMI: Imipramine
IRE1: Inositol requiring enzyme 1
LC3: Light chain 3
LPS: Lipopolysaccharide E. coli
MTEP: 3-[(2-Methyl-1,3-tiazol-4-yl)ethynyl]-pyridine
mTORC1: MTOR complex 1
NS-398: N-[2-(Cyclohexyloxy)-4-itrophenyl]methanesulfonamide
P38MAPK: P38 mitogen-activated protein kinase
PERK: Kinase R (PKR)-like endoplasmic reticulum kinase
PKA: Protein kinase A
PVDF: Methanol-activated polyvinylidene difluoride membranes
ROS: Reactive oxygen species
TFE3: Transcription factor E3
Tisp40: Testis-specific bZip type transcription factor
ULK1: Unc-51-like kinase 1
UPR: Unfolded protein response
