# Supplementary material for: Elucidating the molecular mechanisms underlying the induction of autophagy by antidepressant-like substances in C57BL/6J mouse testis model upon LPS challenge
Source: Cell Commun Signal. 2023 Sep 21;21:251. doi: 10.1186/s12964-023-01270-6 (PMC10512556; doi:10.1186/s12964-023-01270-6)

**cATF6**

12

10 11

9

8

7

1

3 4

6

-

LPS

- C

– MTEP

– NS-398

- IMI

- MTEP+NS-398

– IMI+NS-398

+

7

8

9

1

1

1

LPS

– C

– MTEP

– NS-398

0 – IMI

1 – MTEP+NS-398

2 – IMI+NS-398

1

2

3

4

5

6


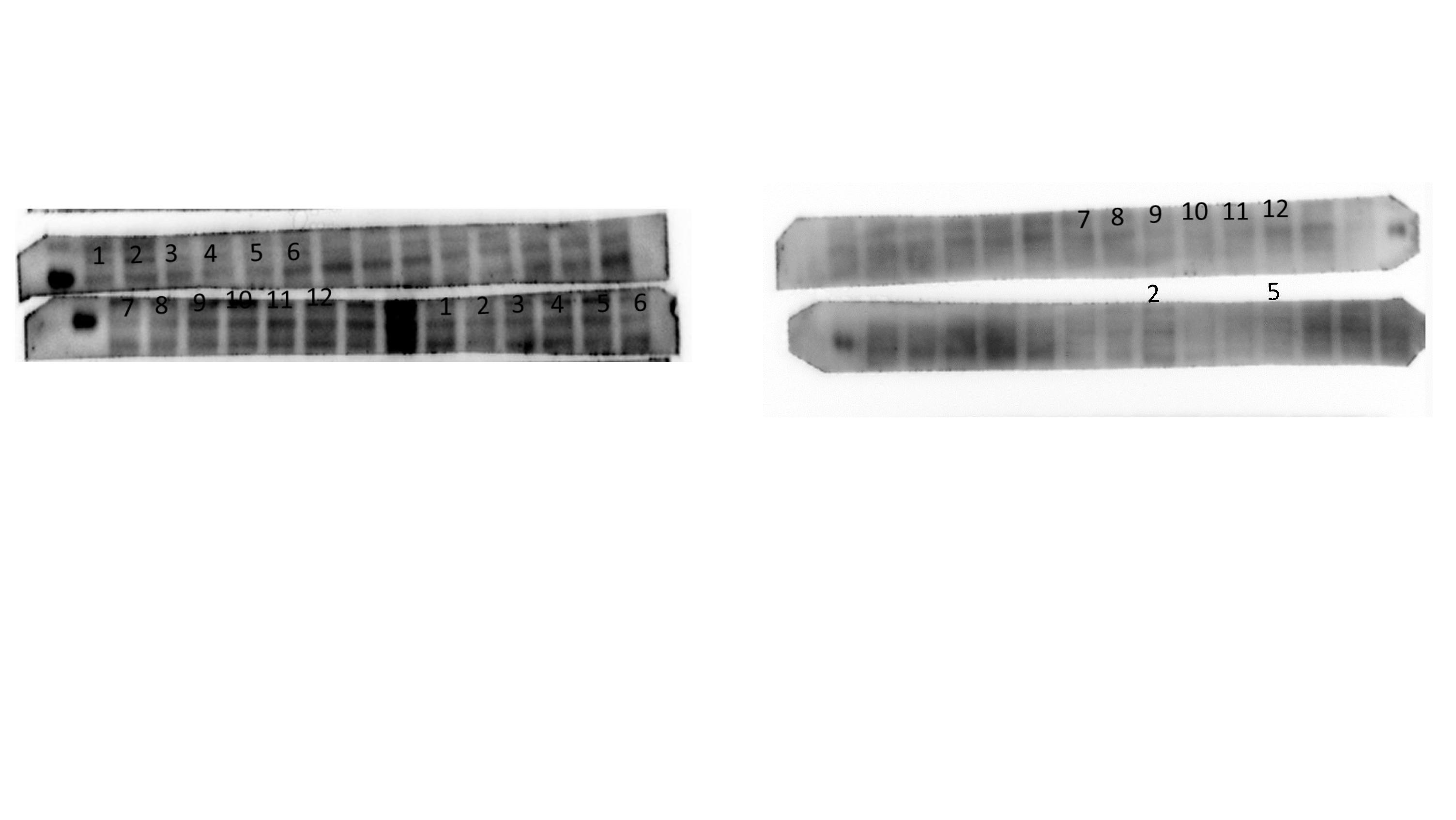


p-PERK

6

4

5


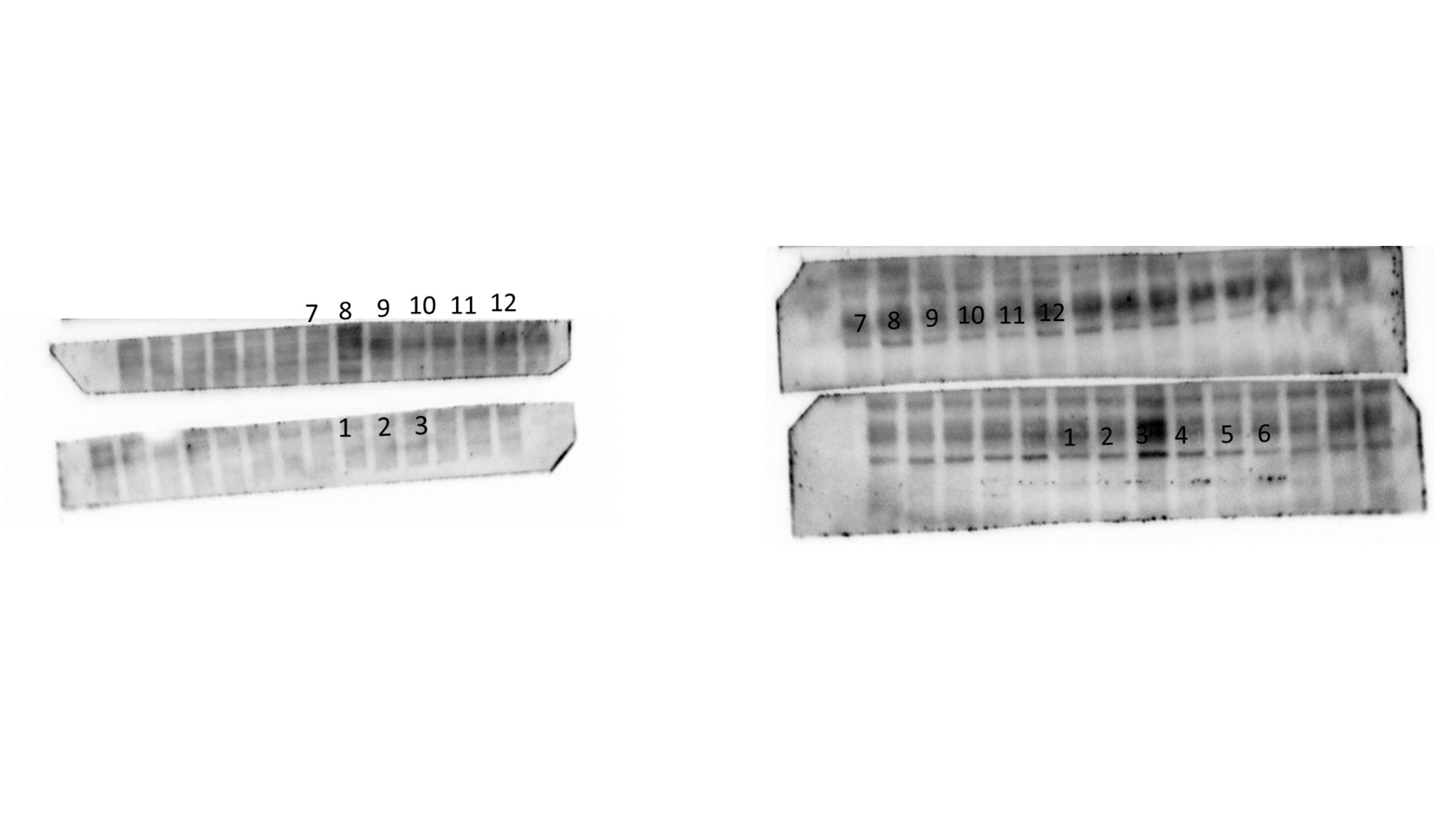


p-IRE

11 12

10

7

8

9


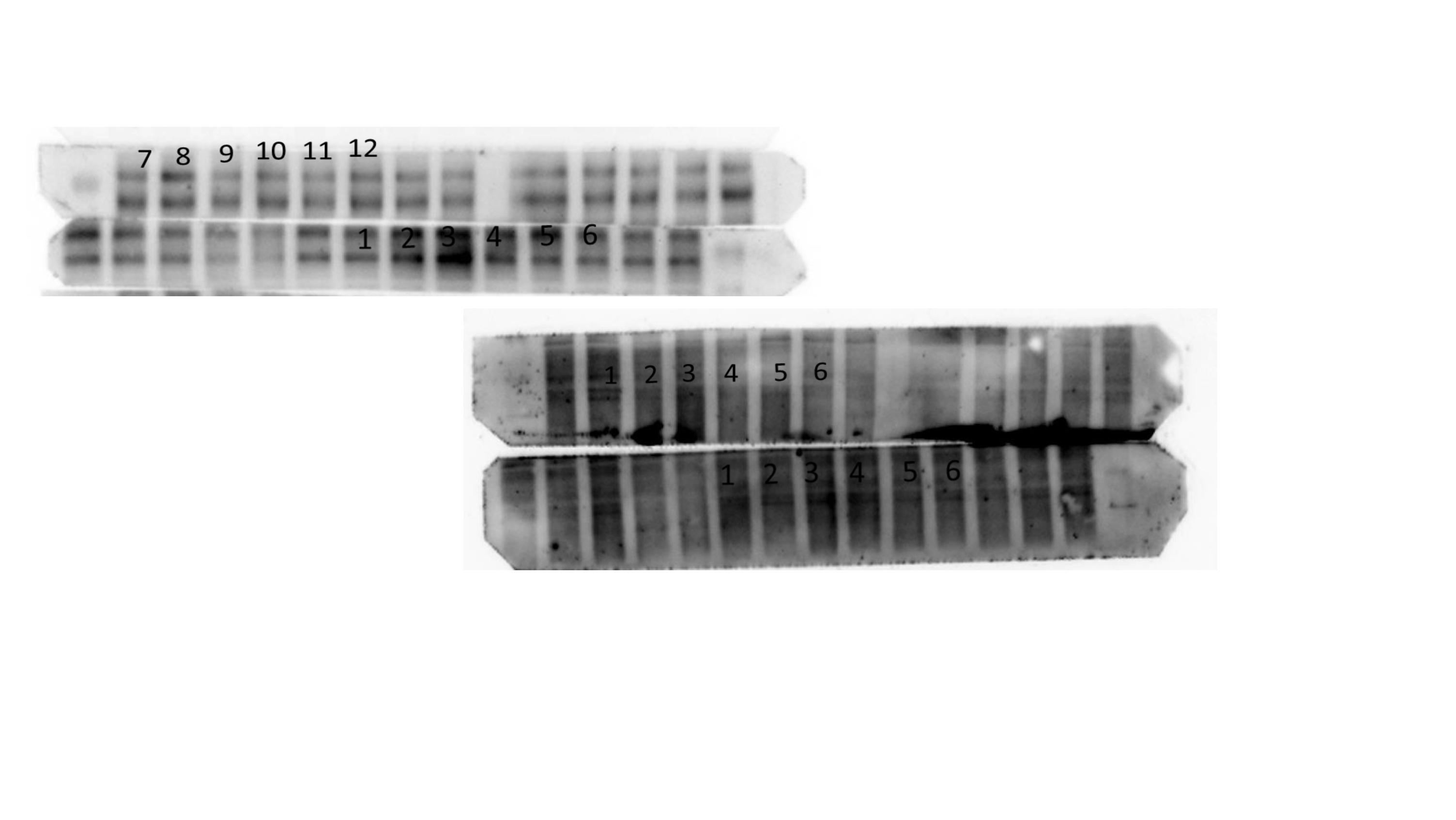


p-ASK1

7

8

9

10 11 12

5 6

4


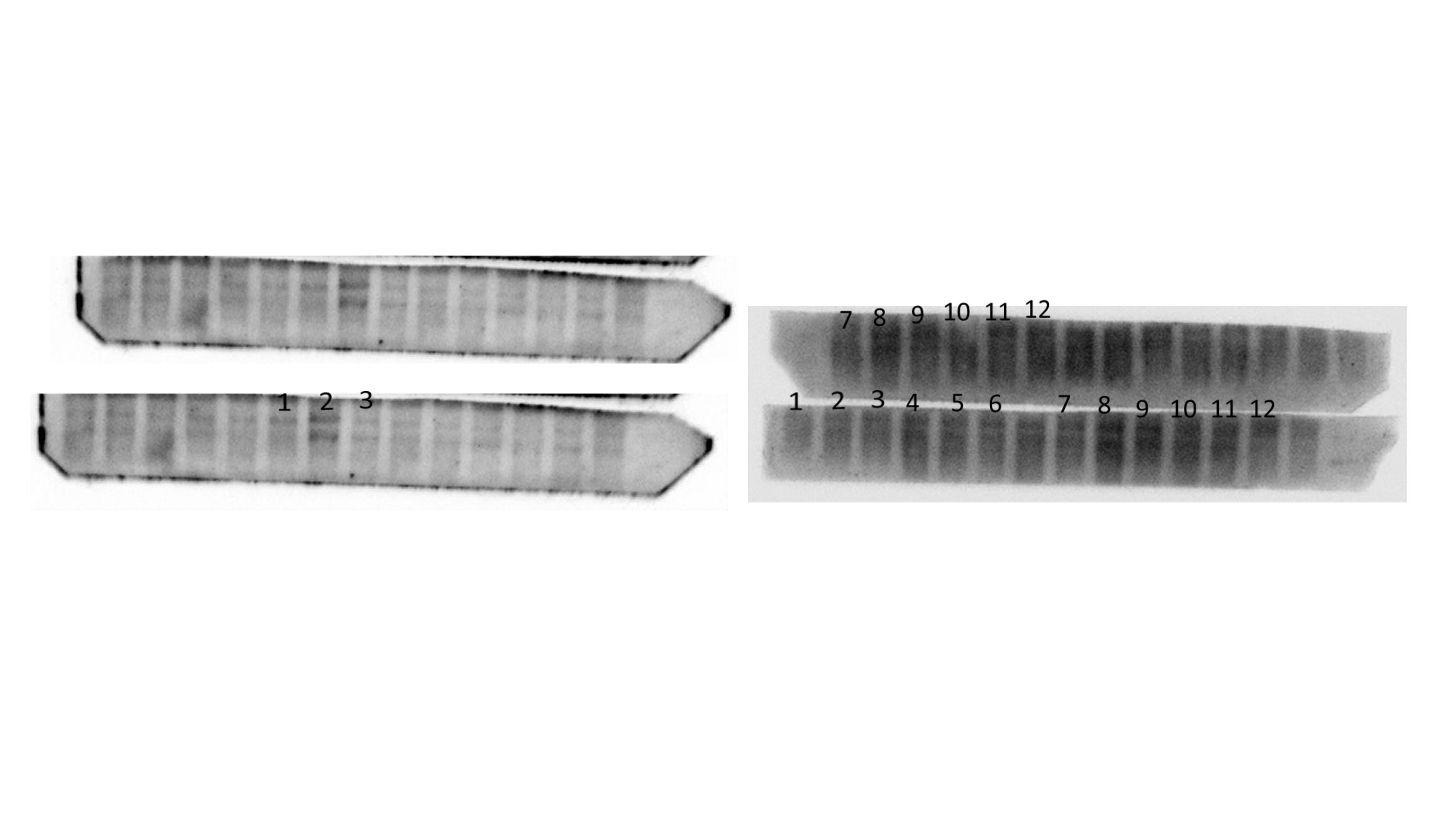


TRAF2

4

5 6


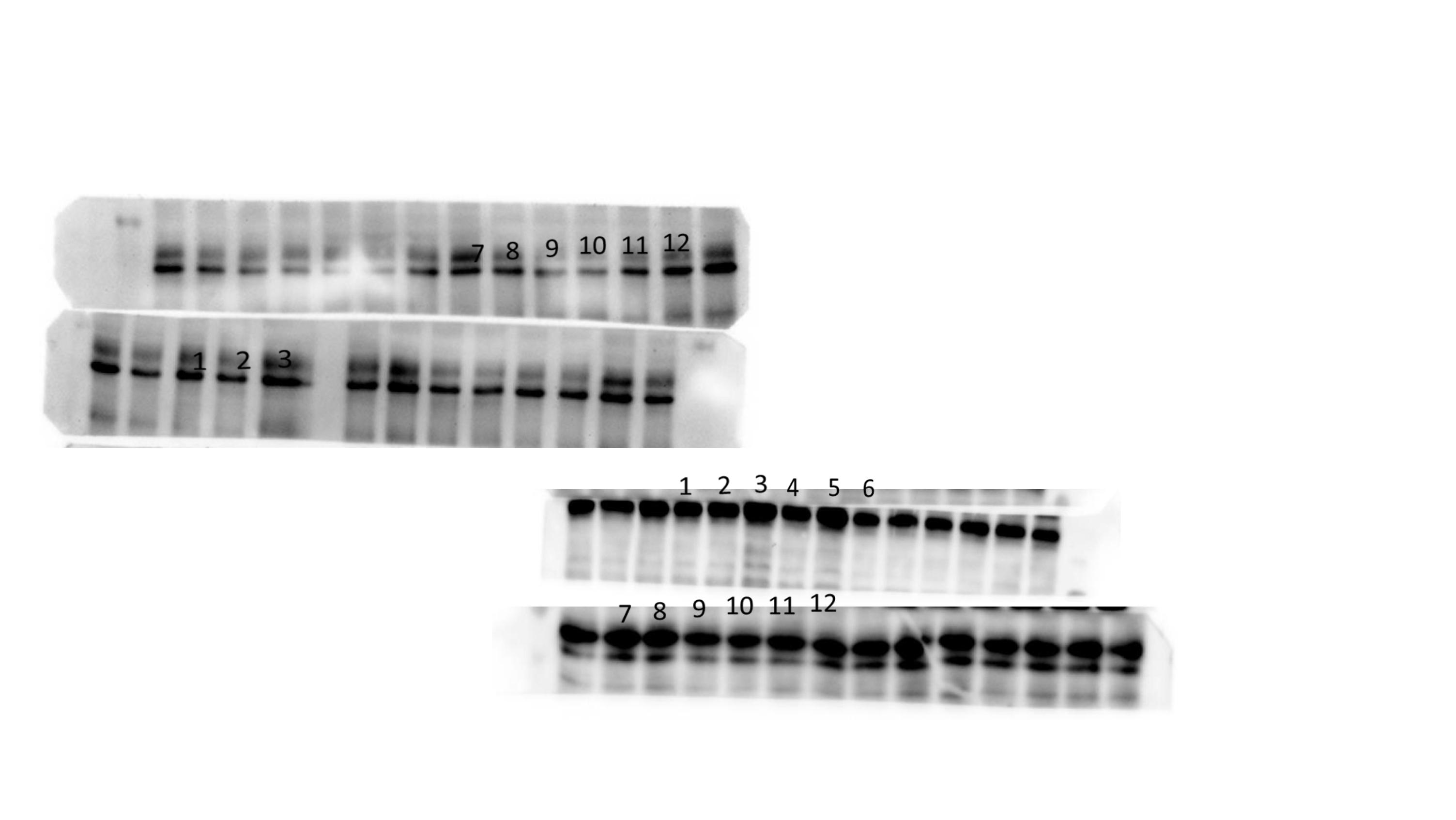


p-p38MAPK


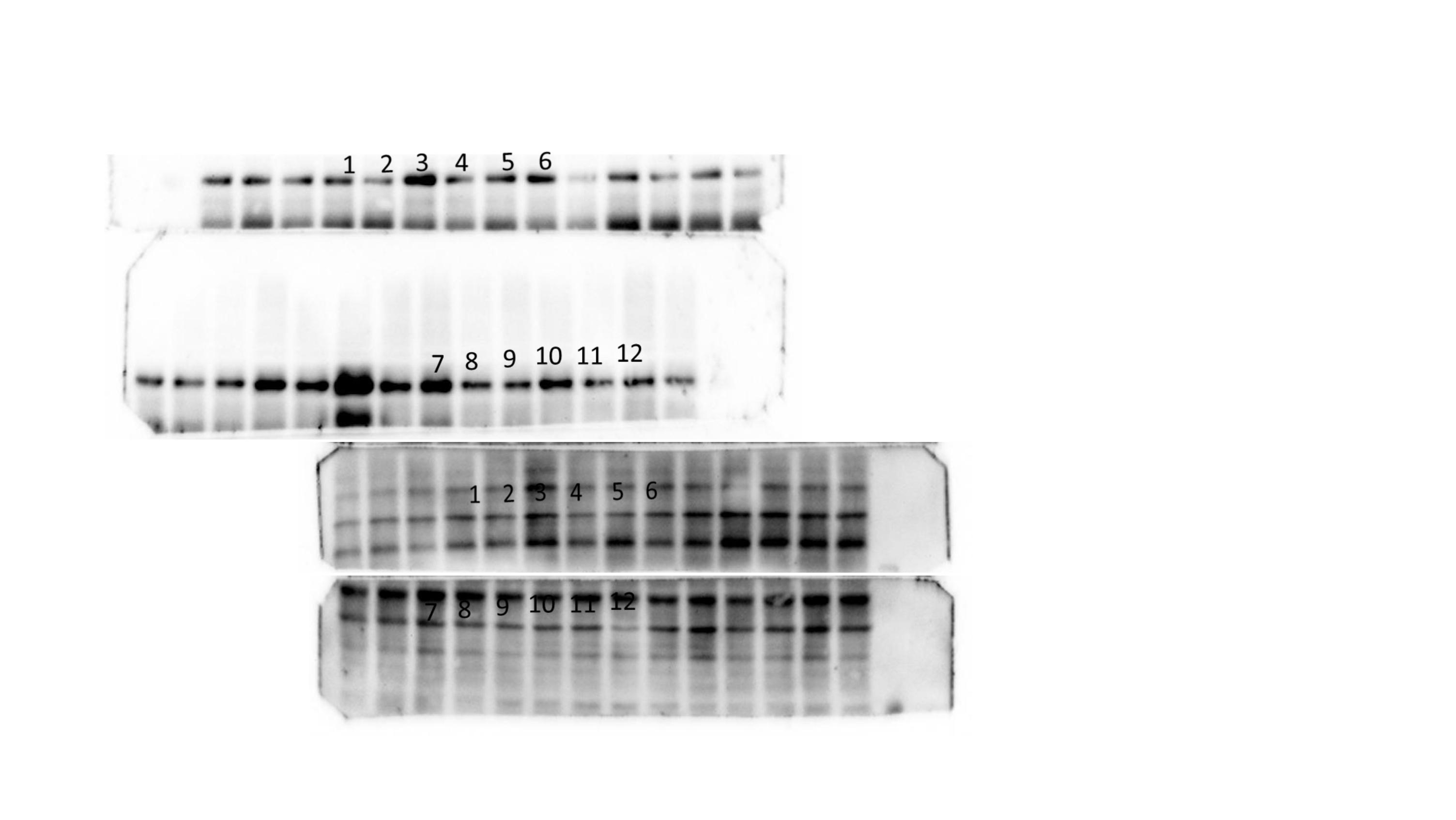


p-eiF2α


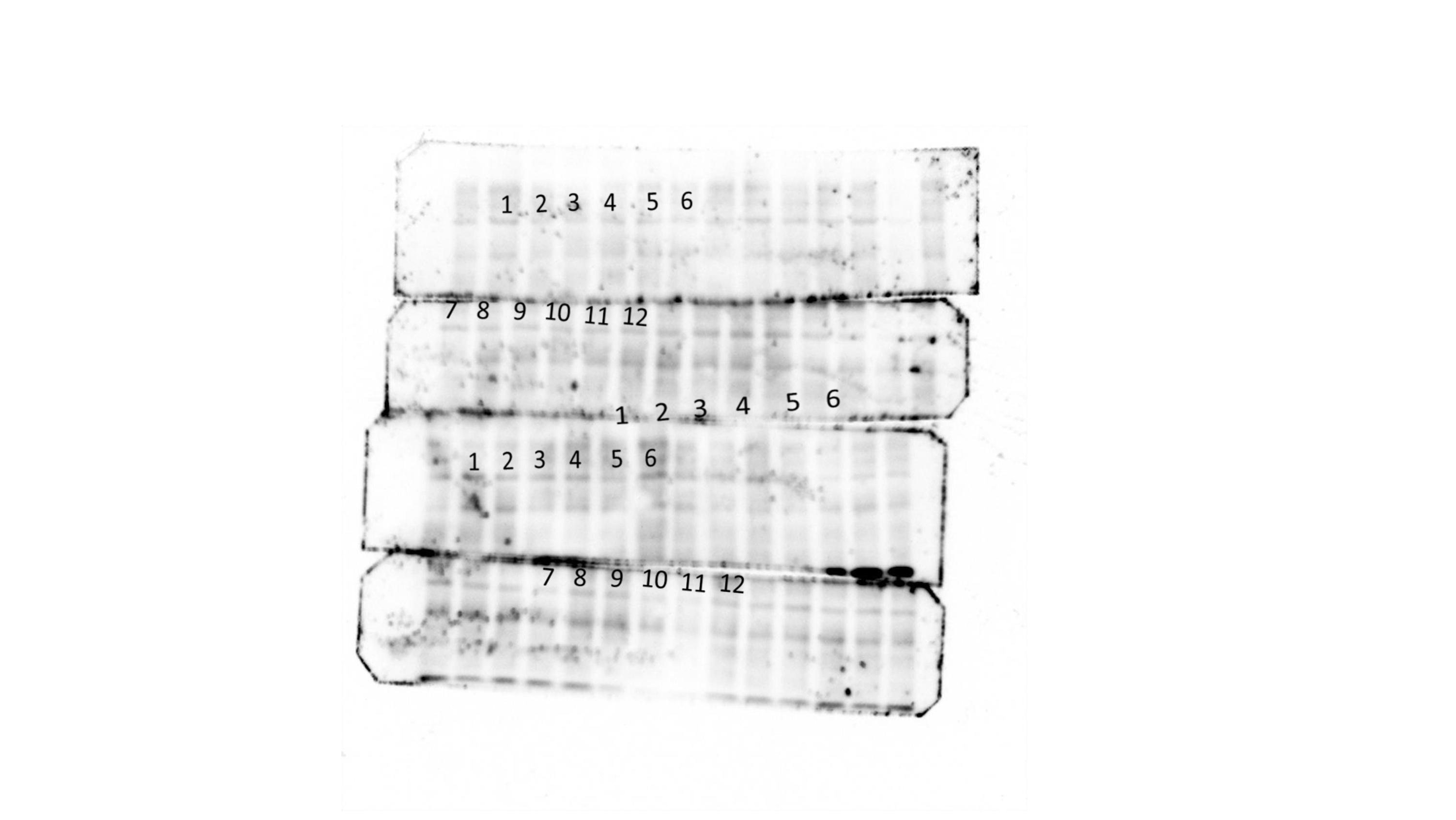


P-ATF4

8

7

10 9

12 11


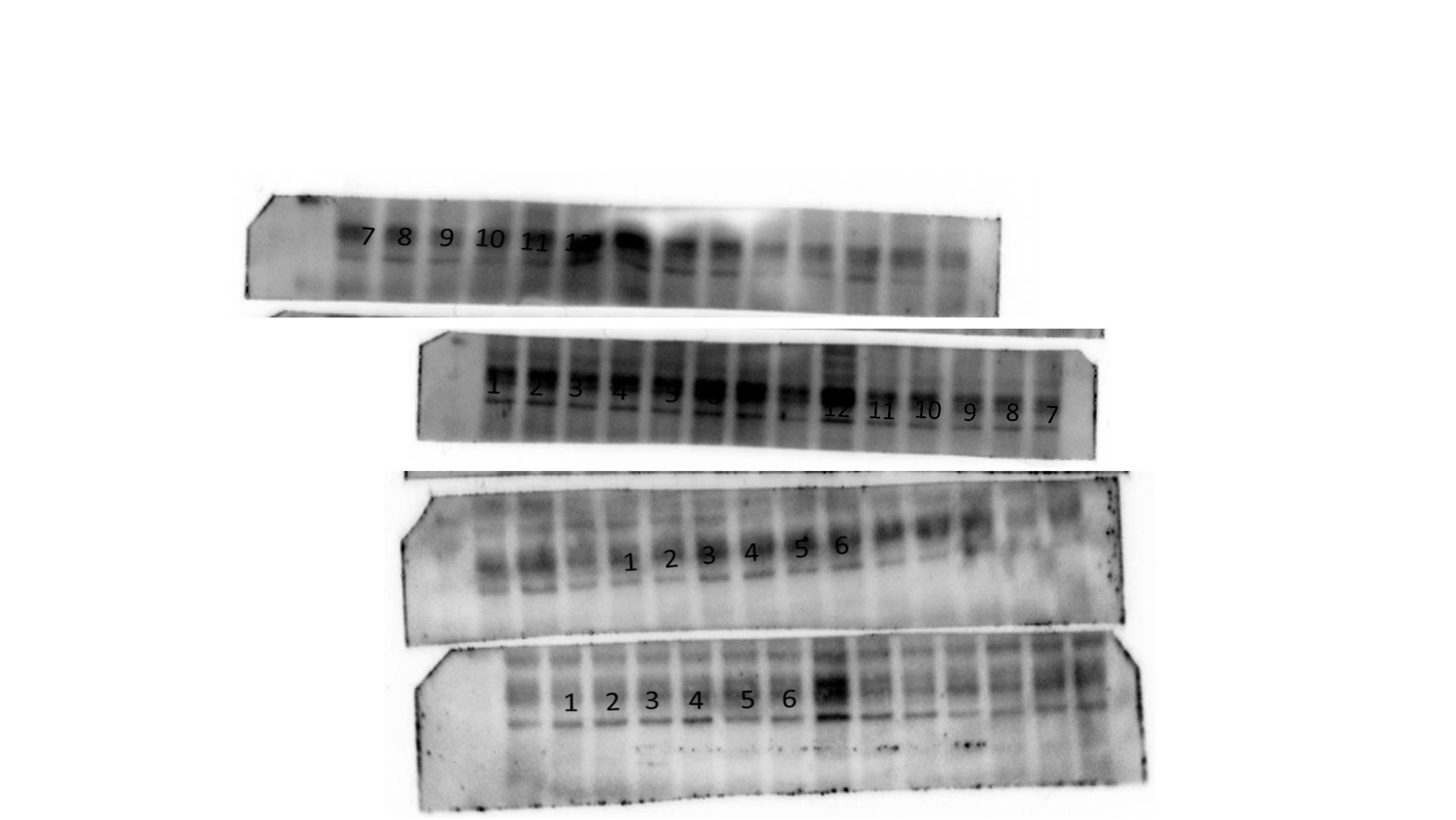


P-CHOP


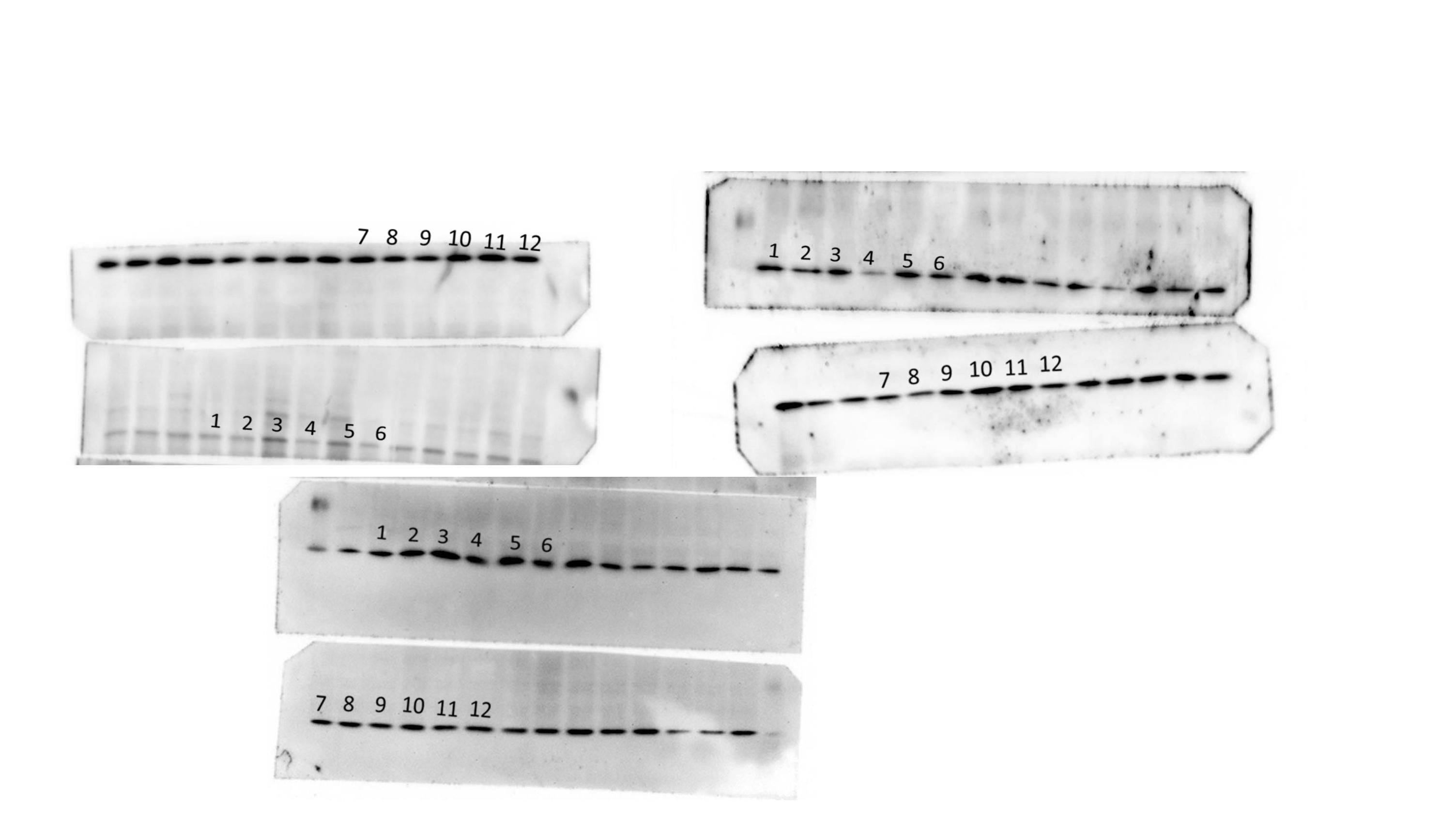


GADD34


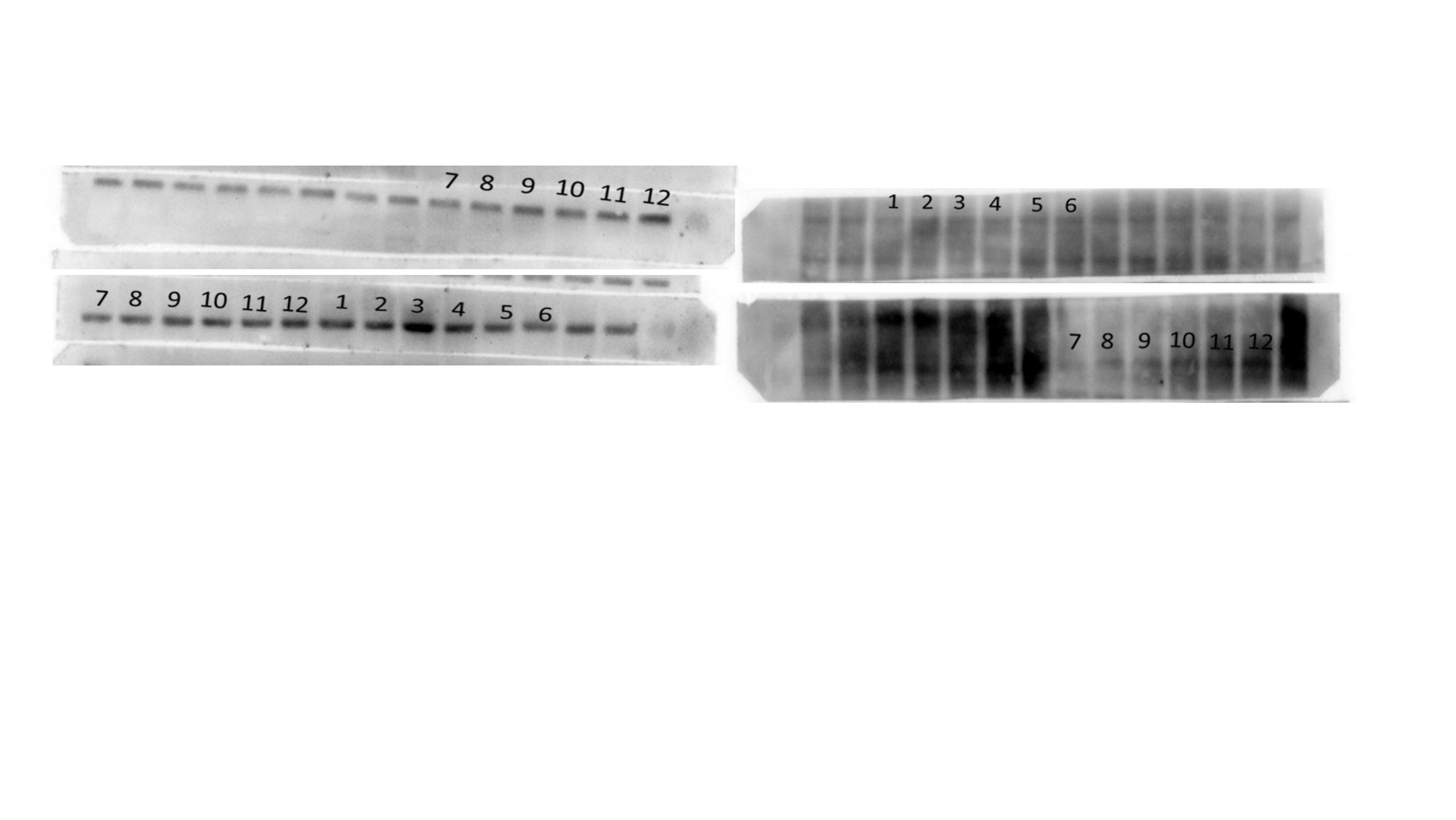


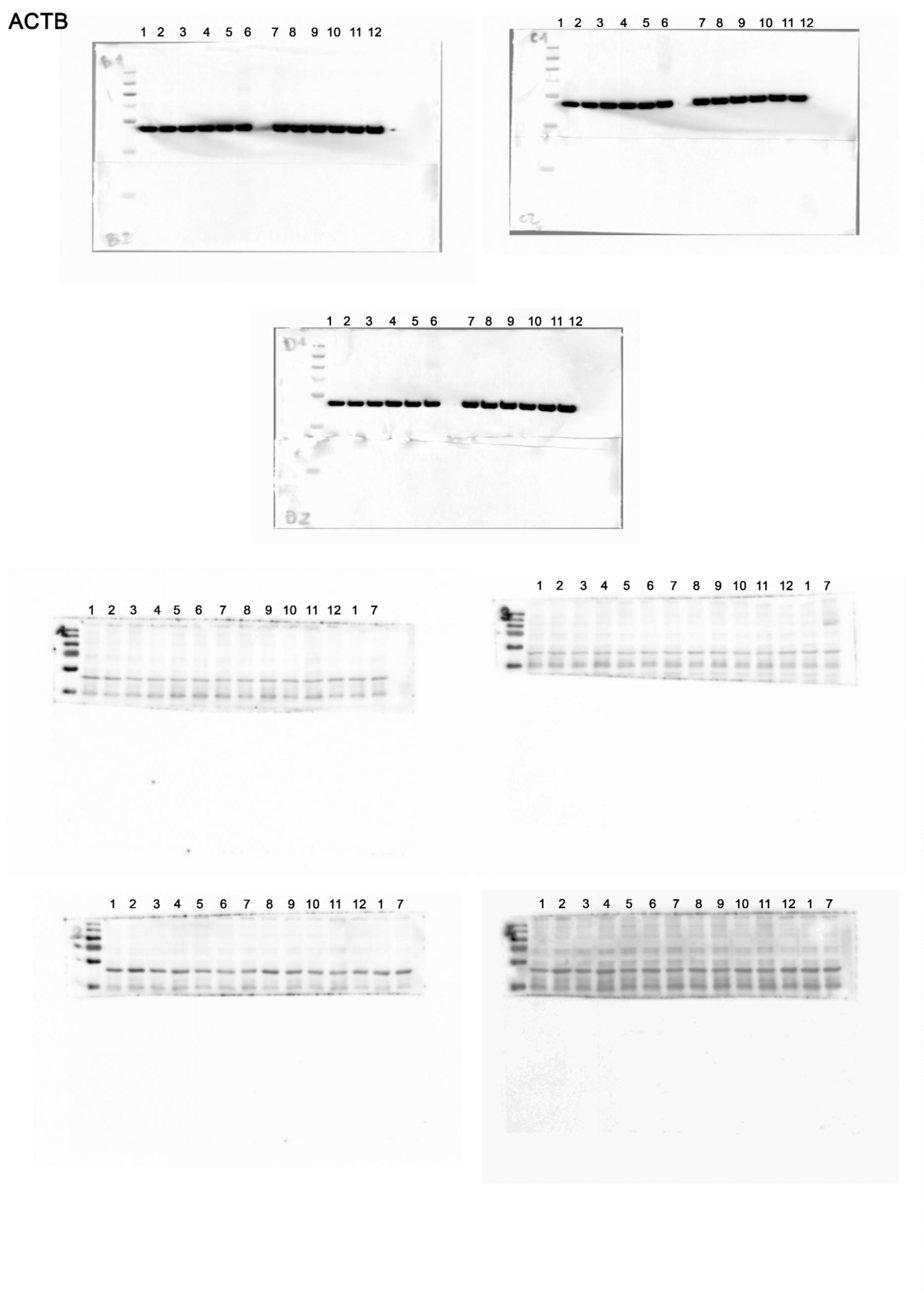


mTOR

7

8 9

10 11 12

1

2 3 4

5

6


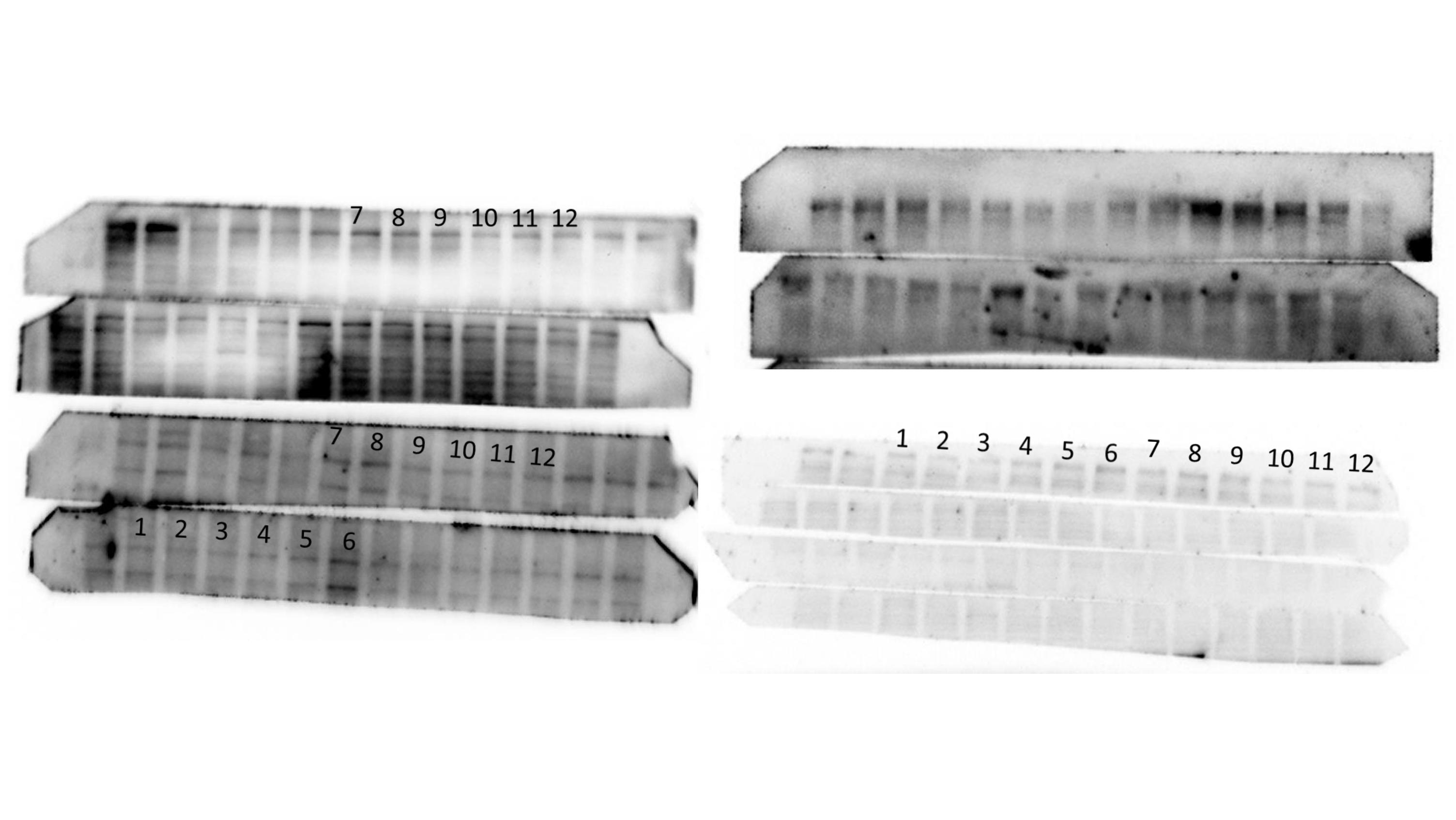


ULK1

11 12

7

8 9 10


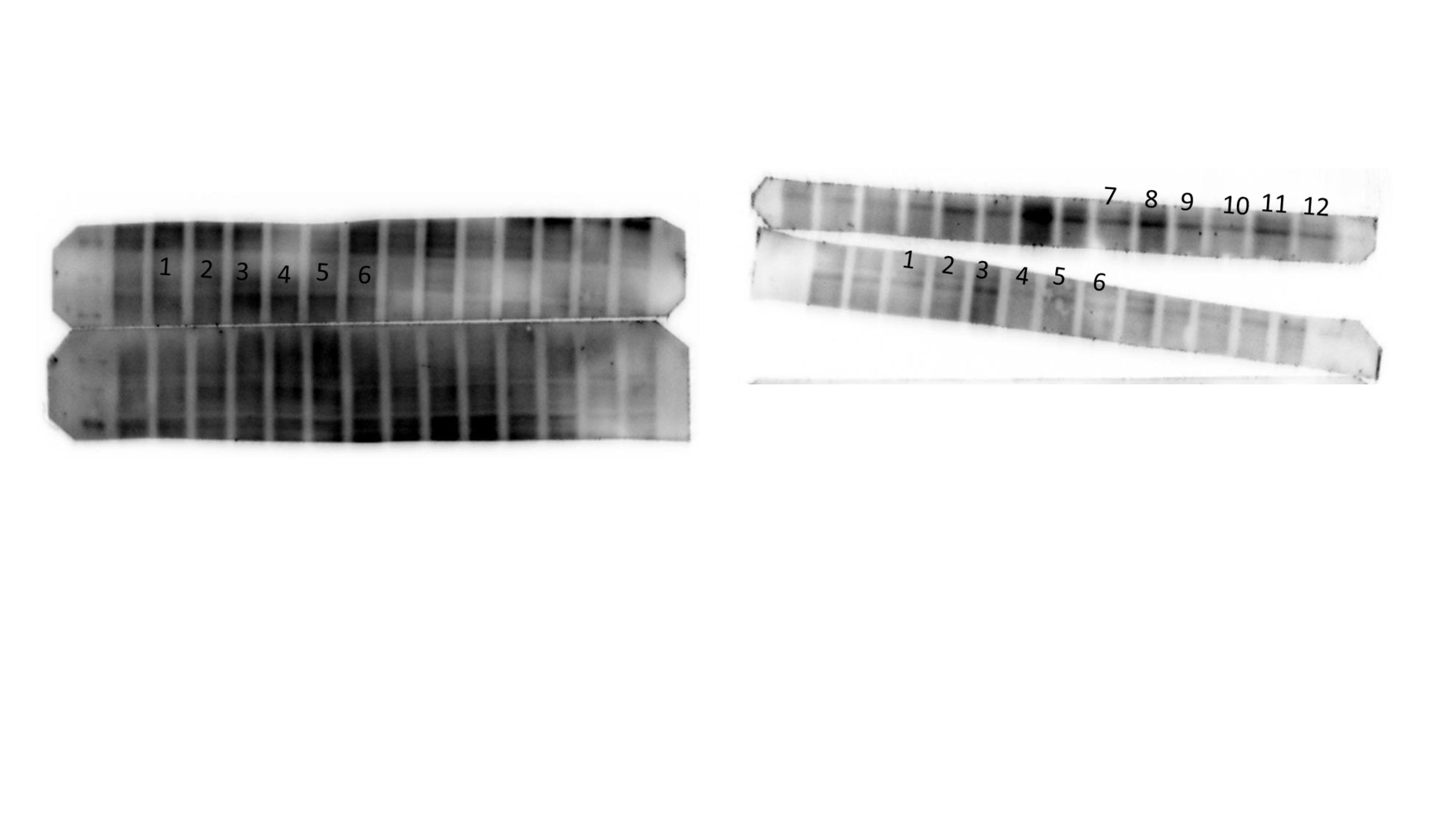


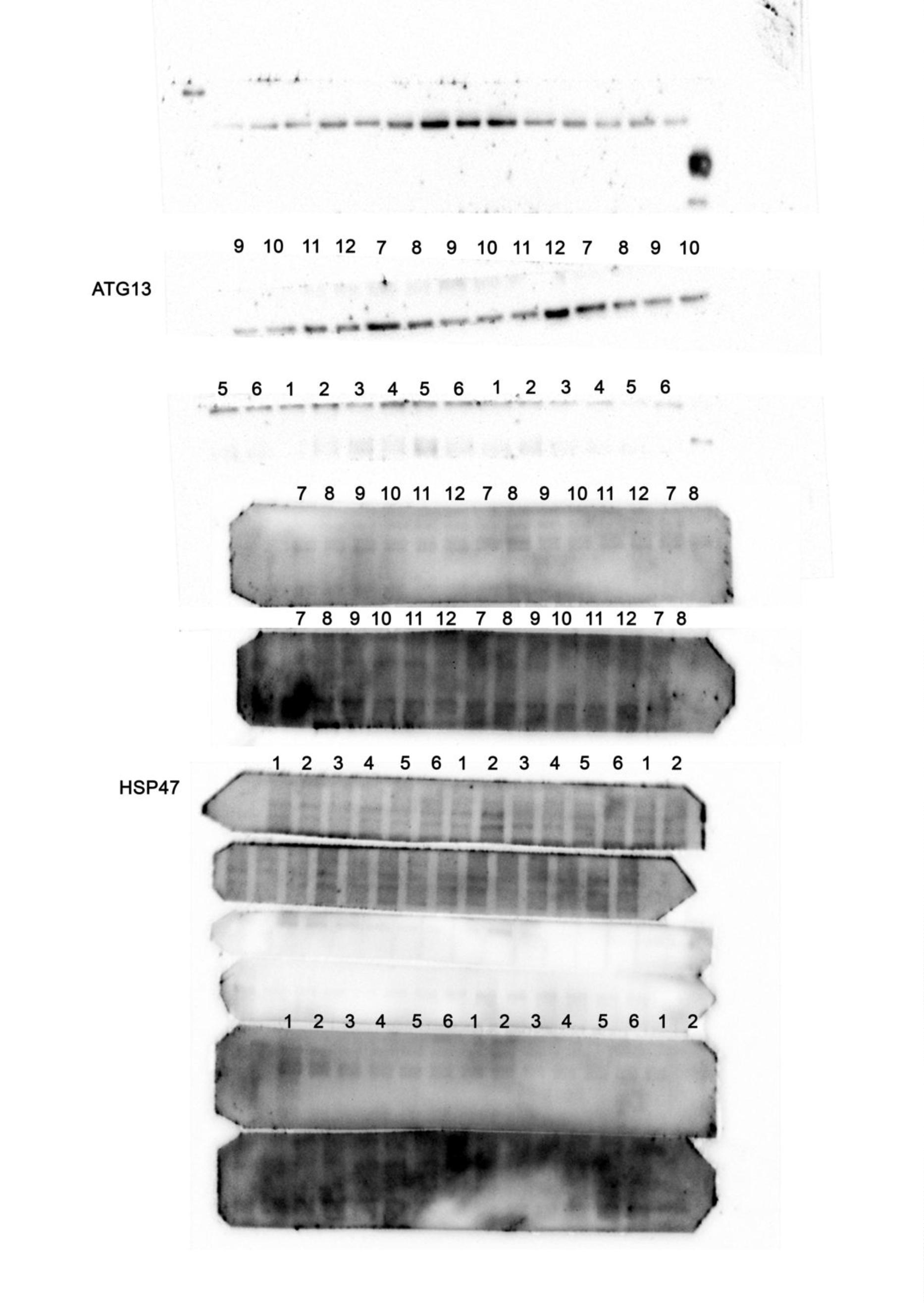


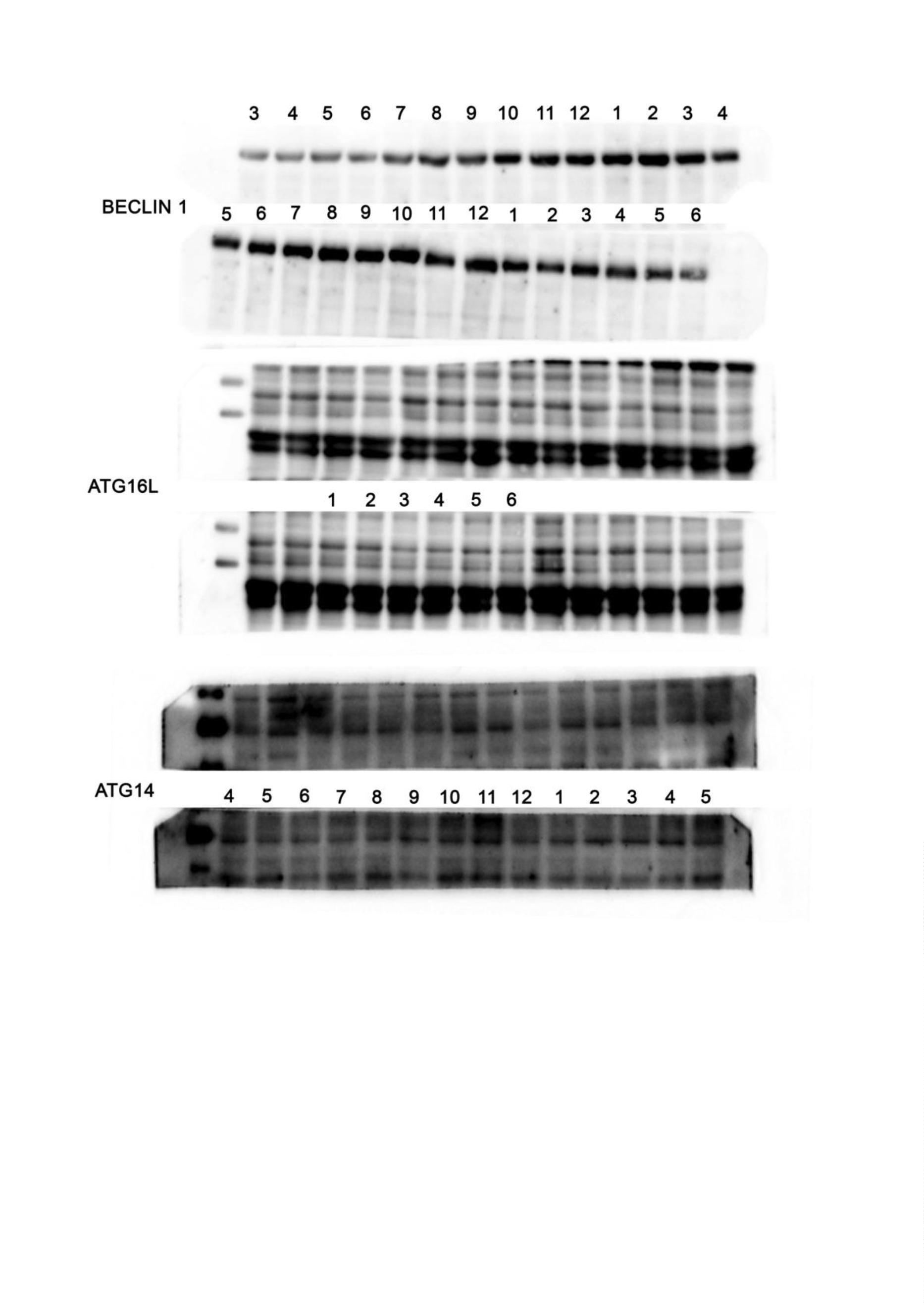


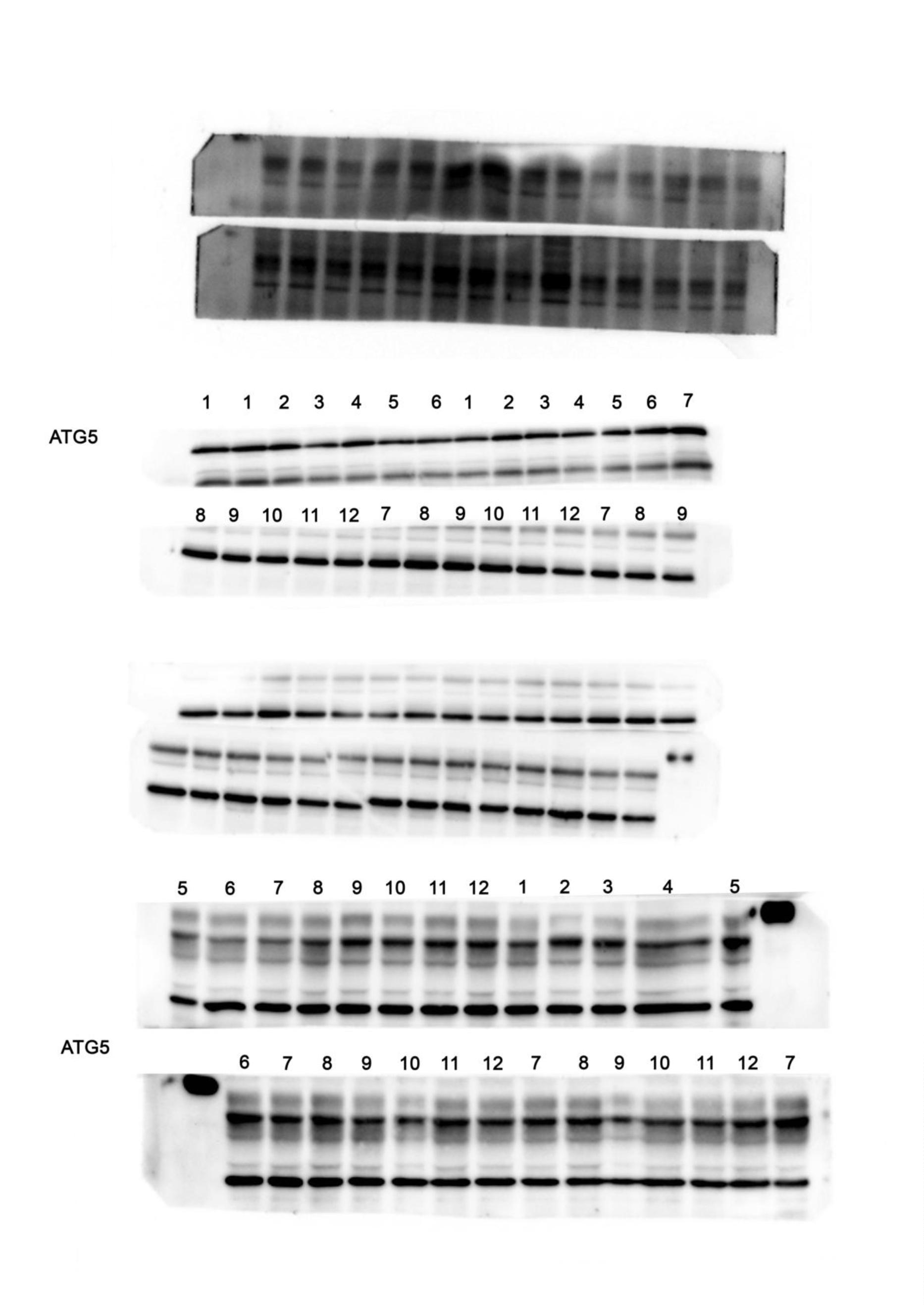


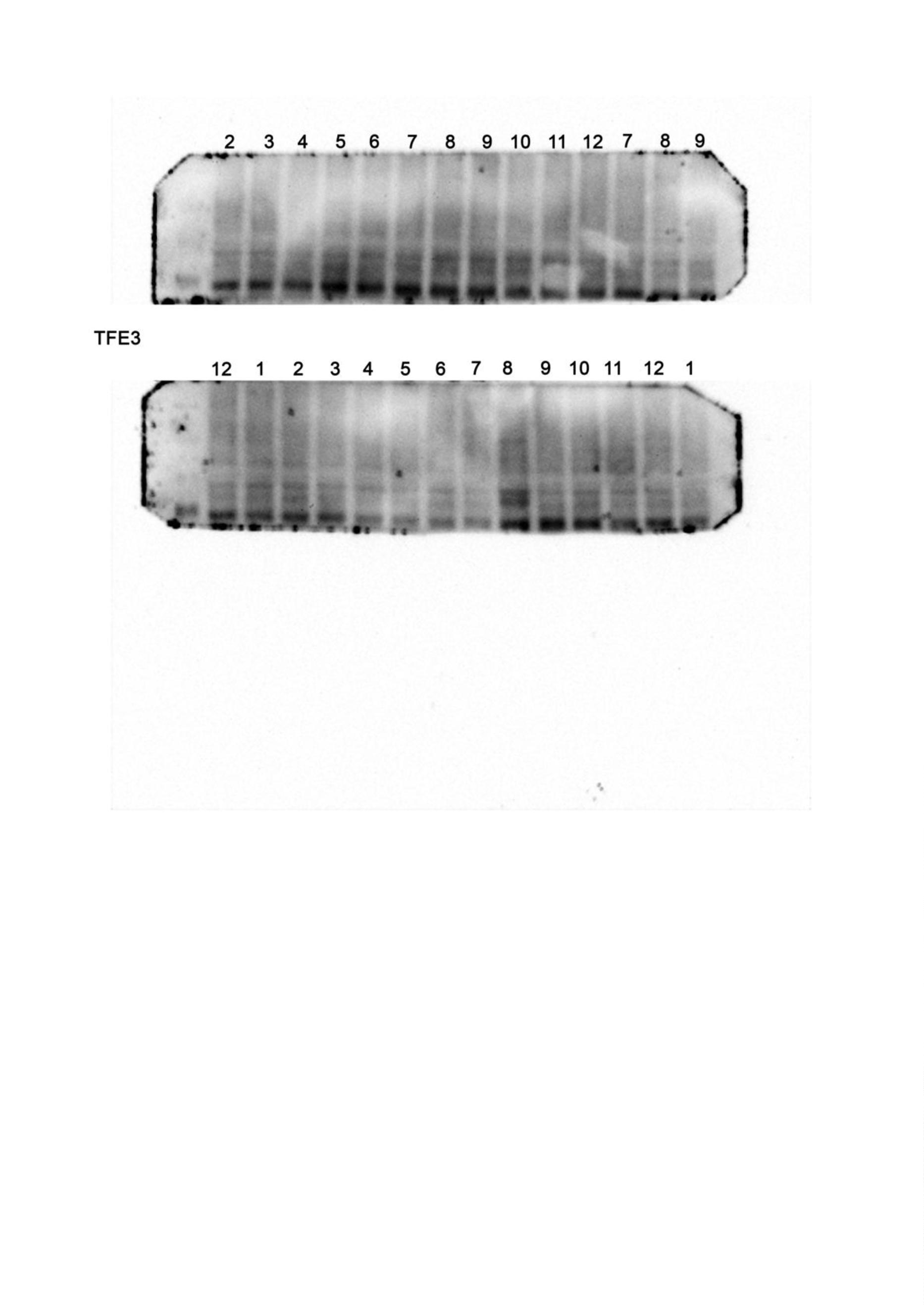


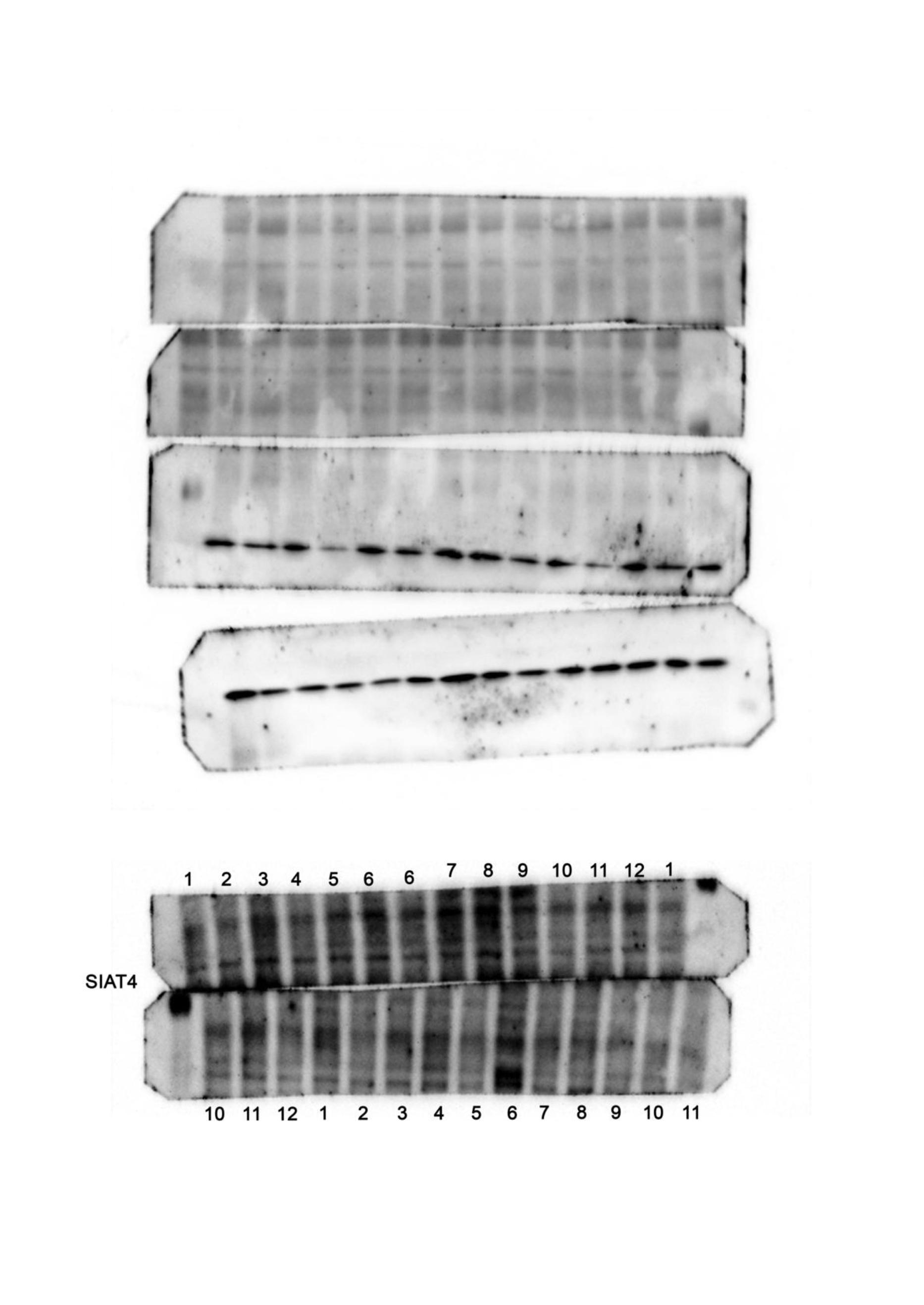


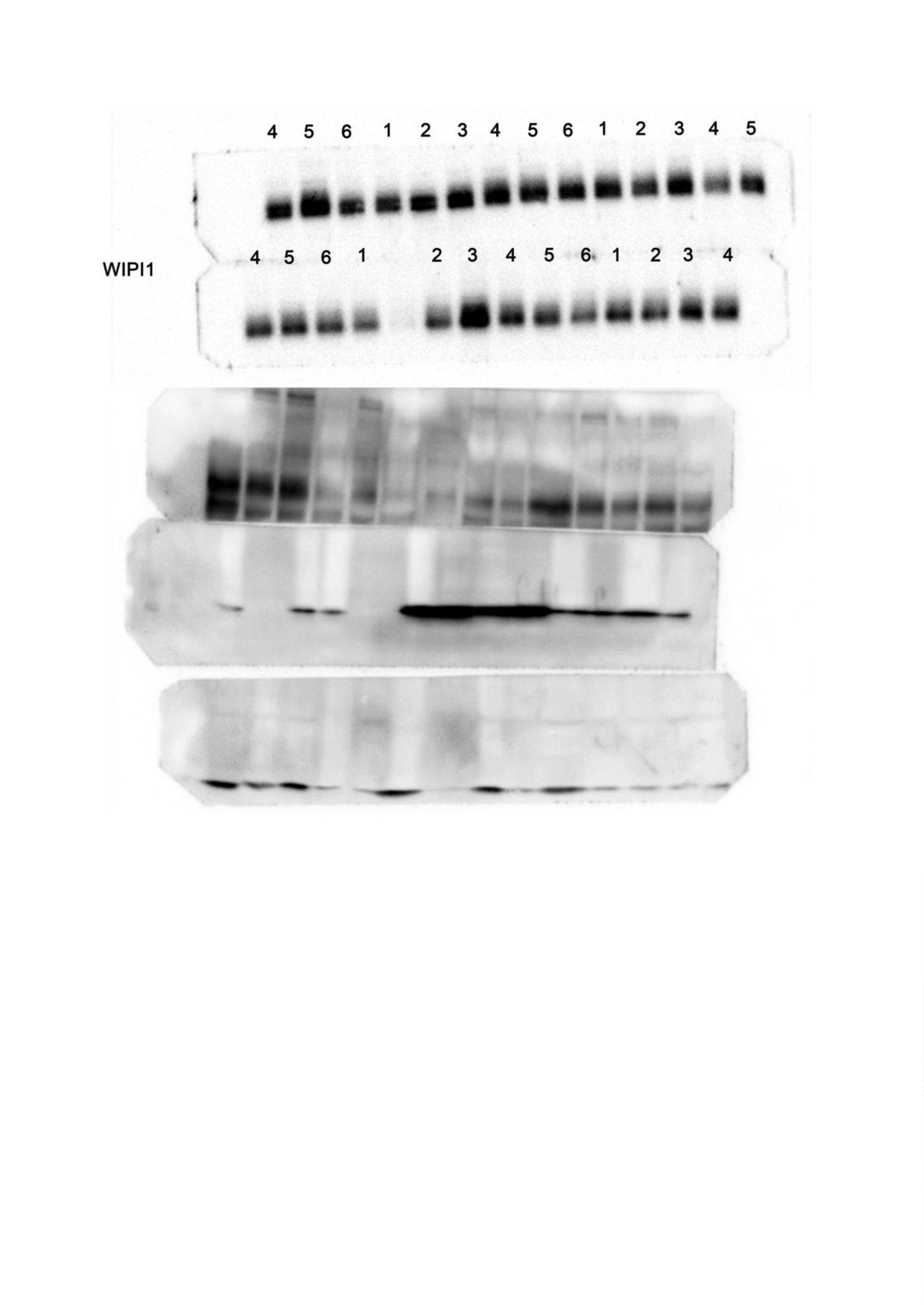


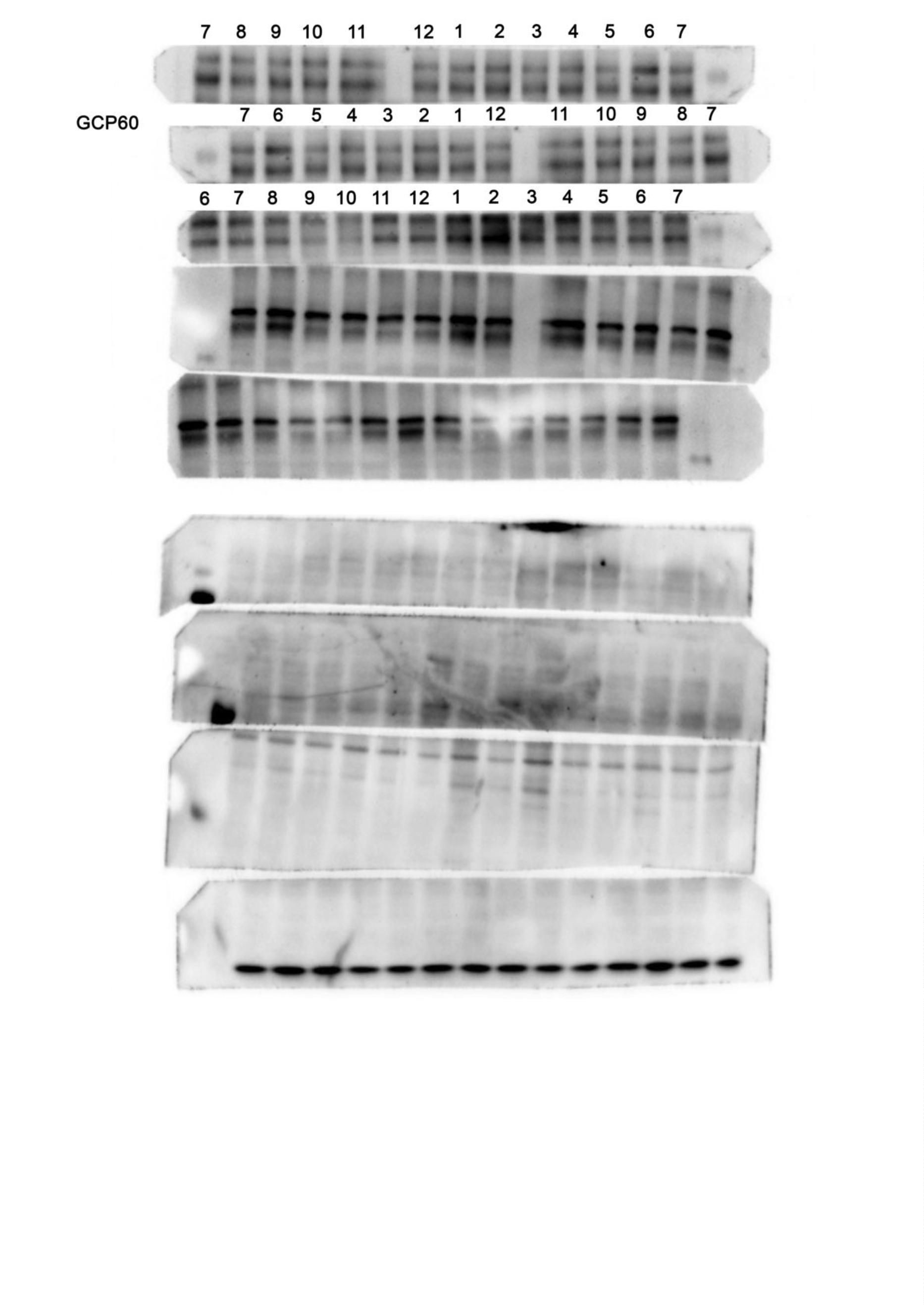


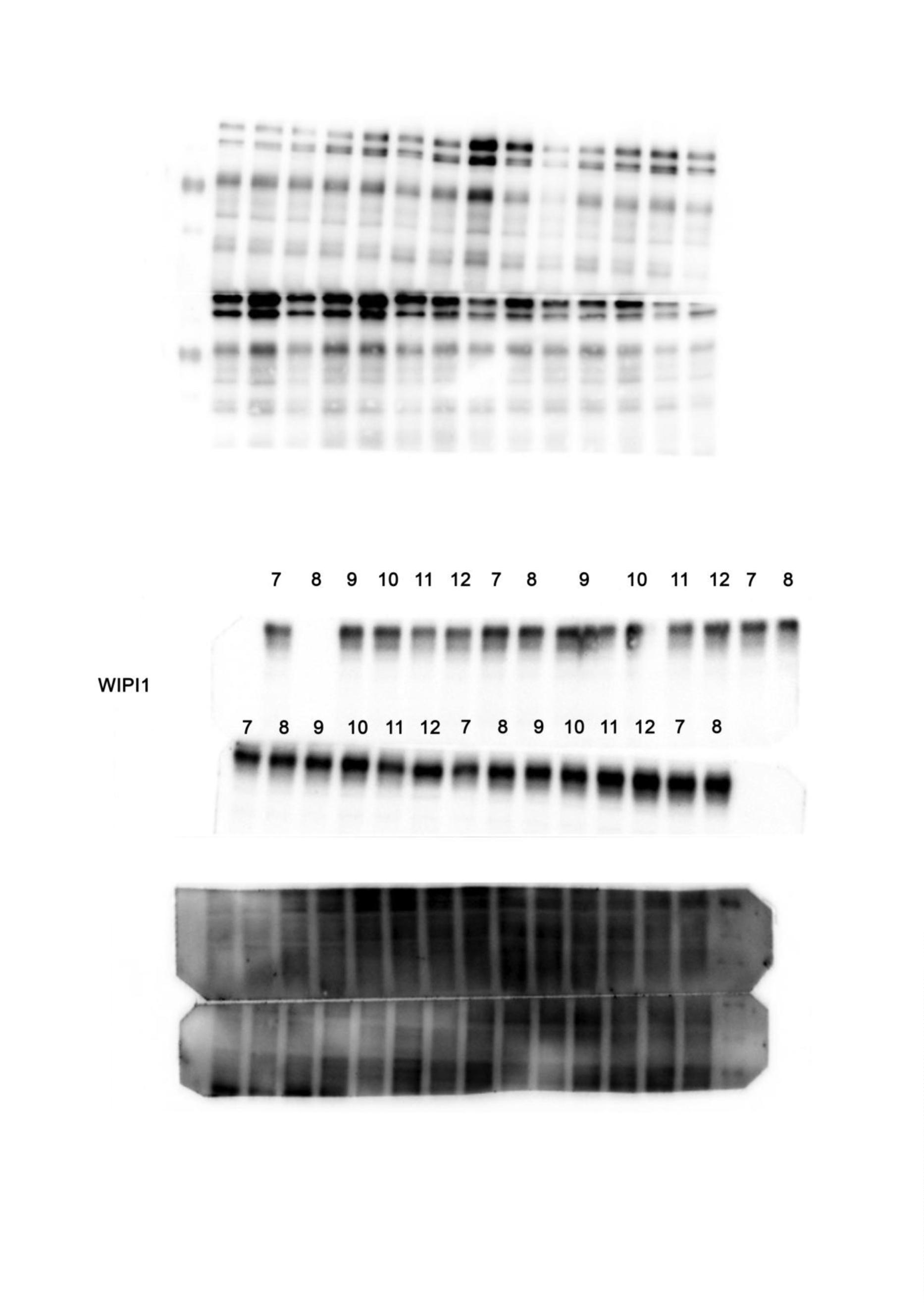


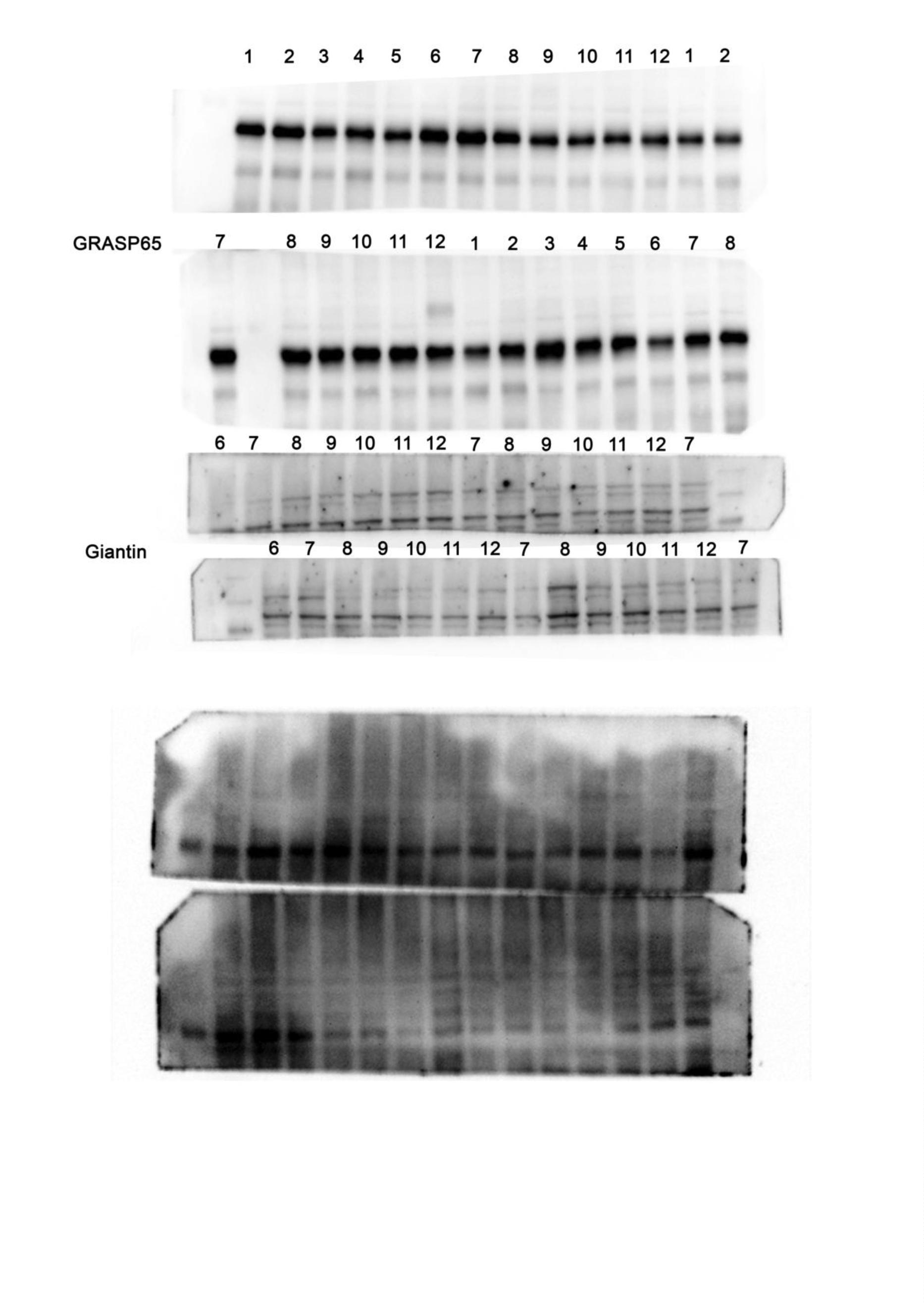


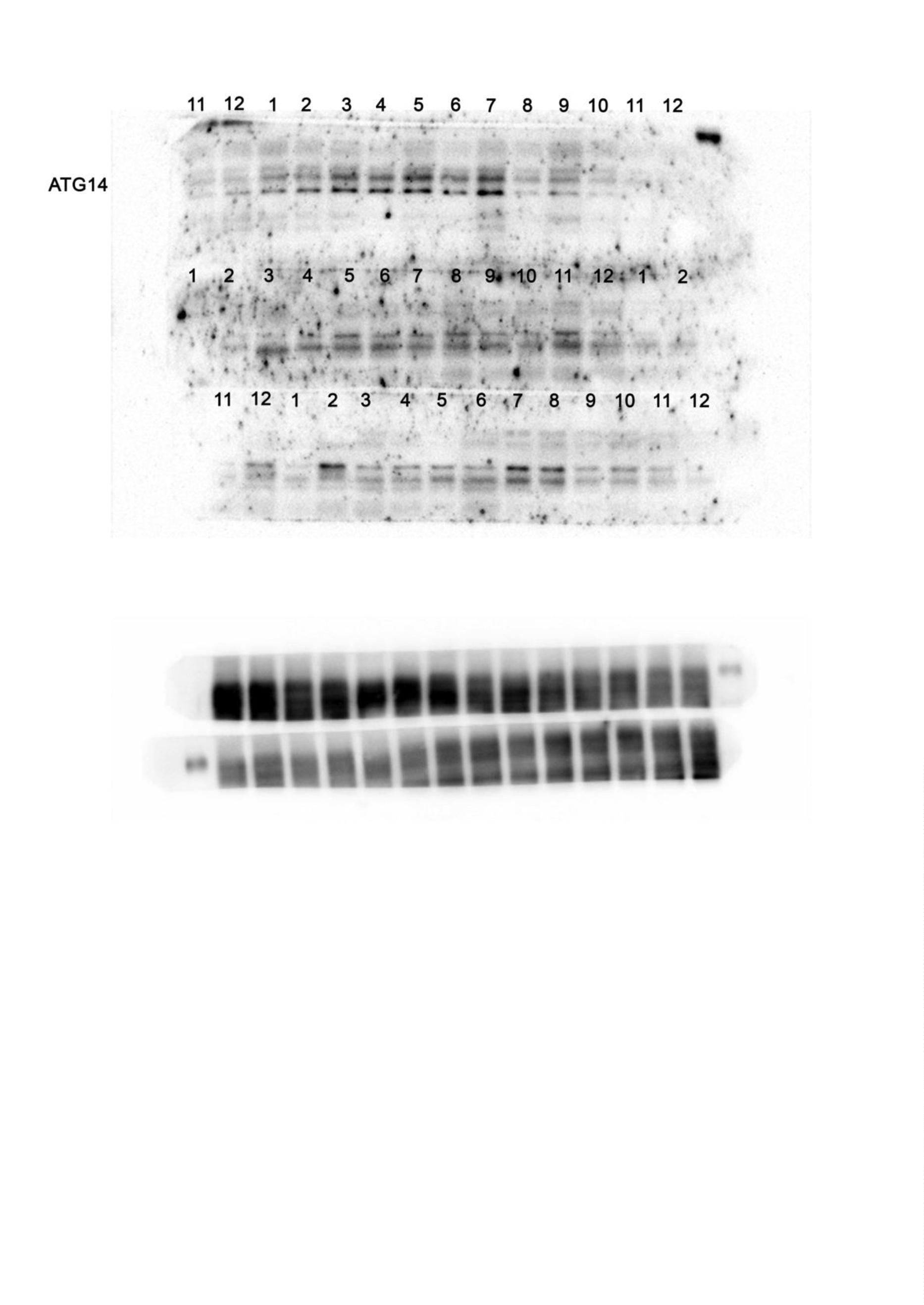


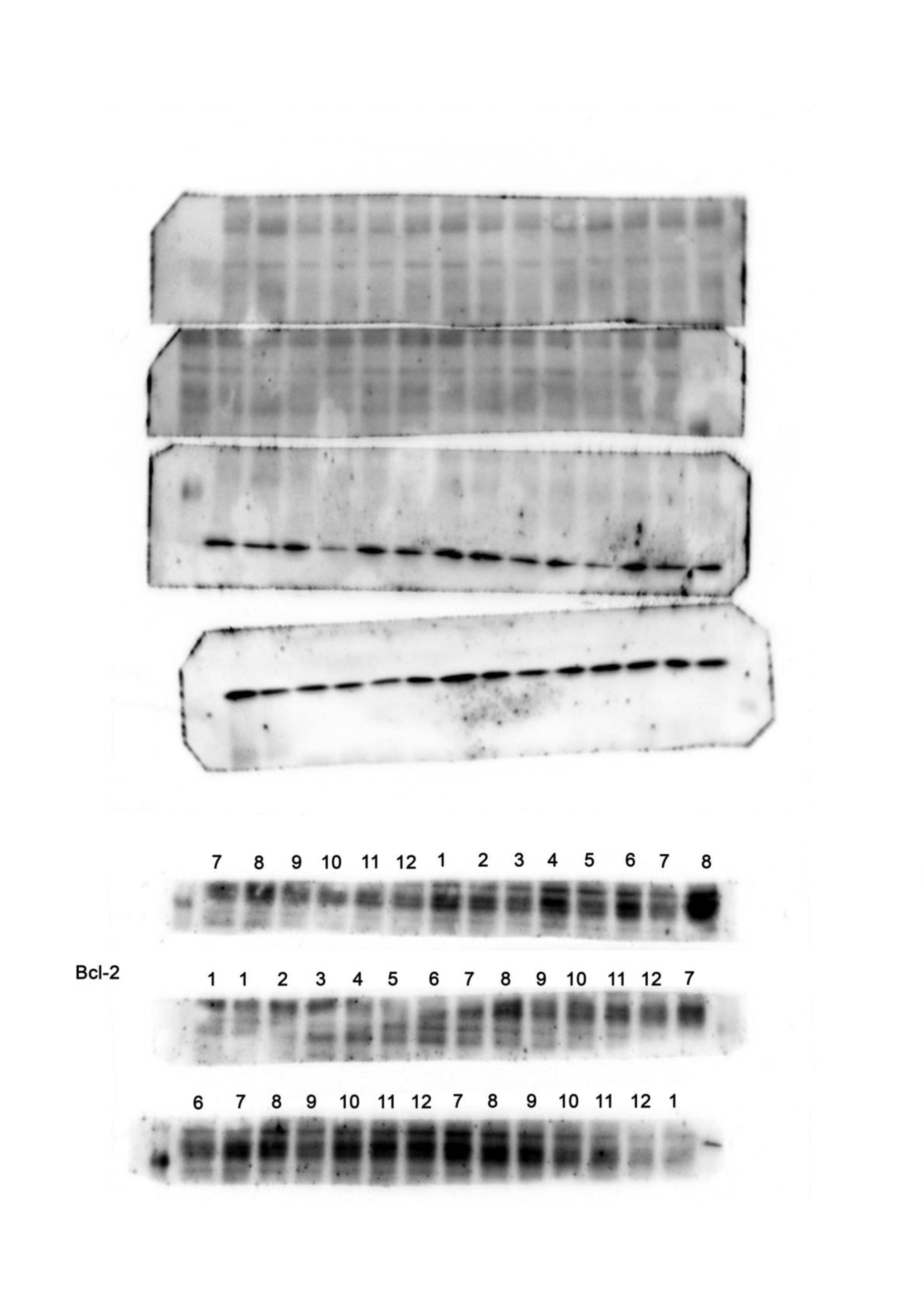


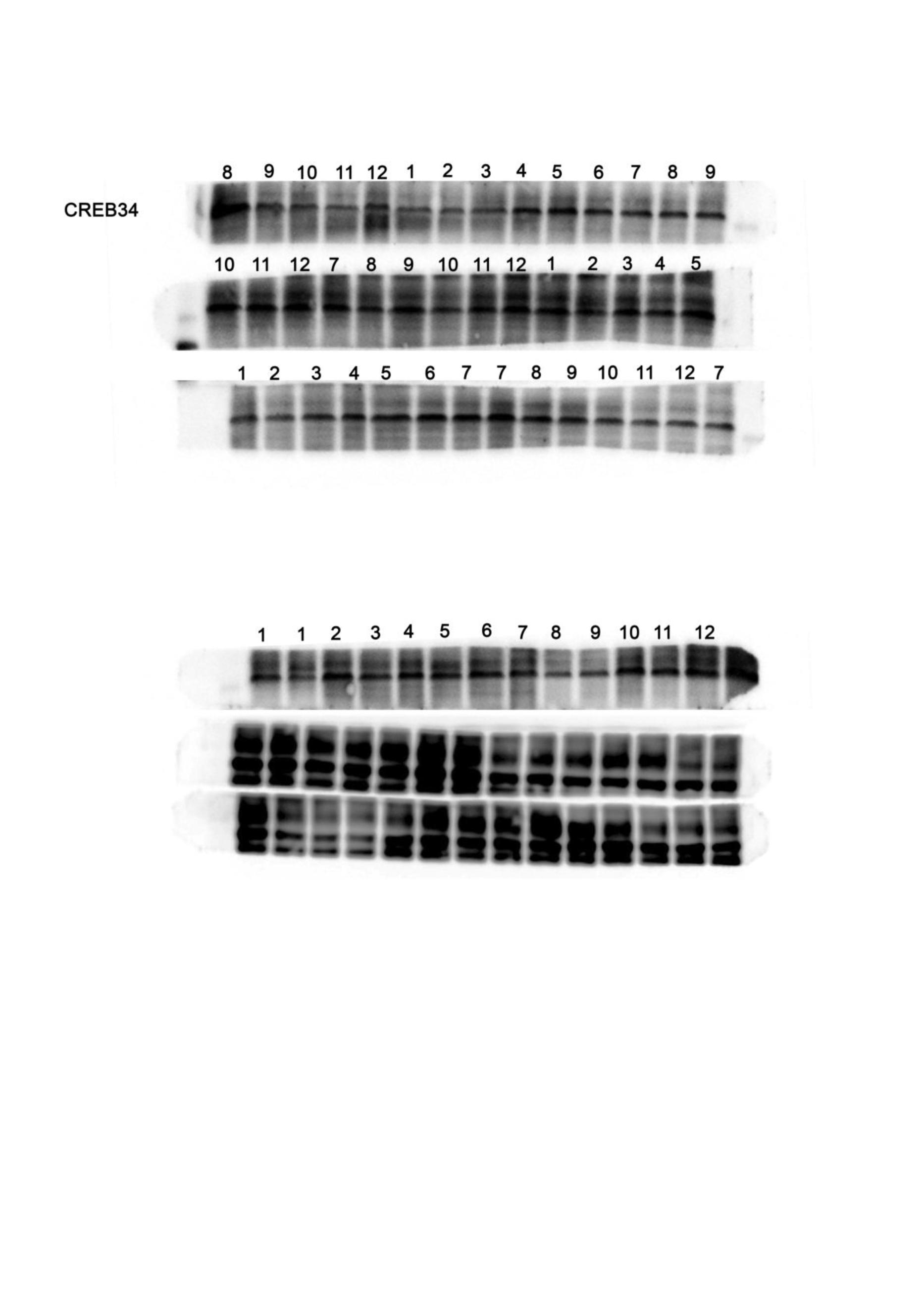


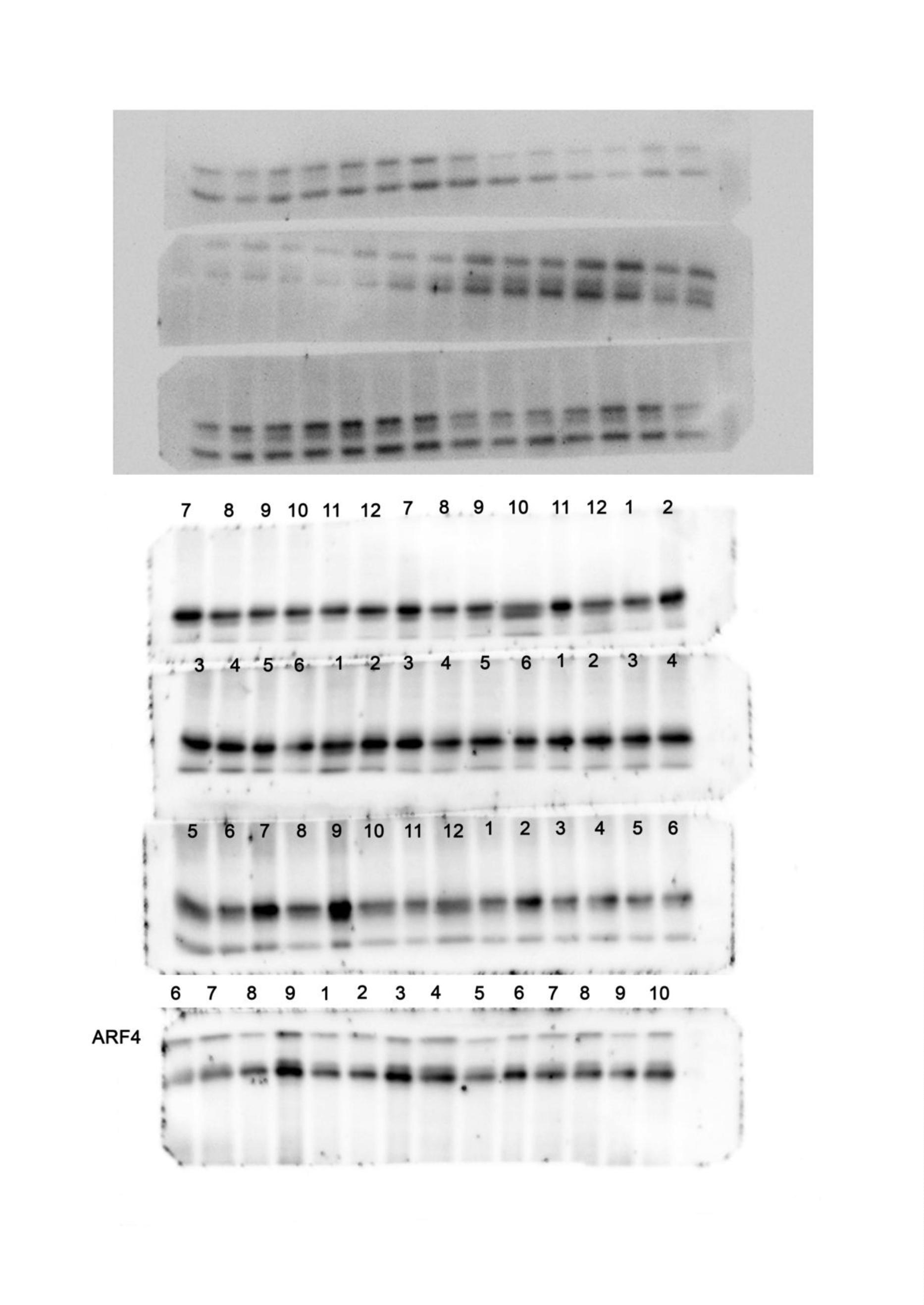


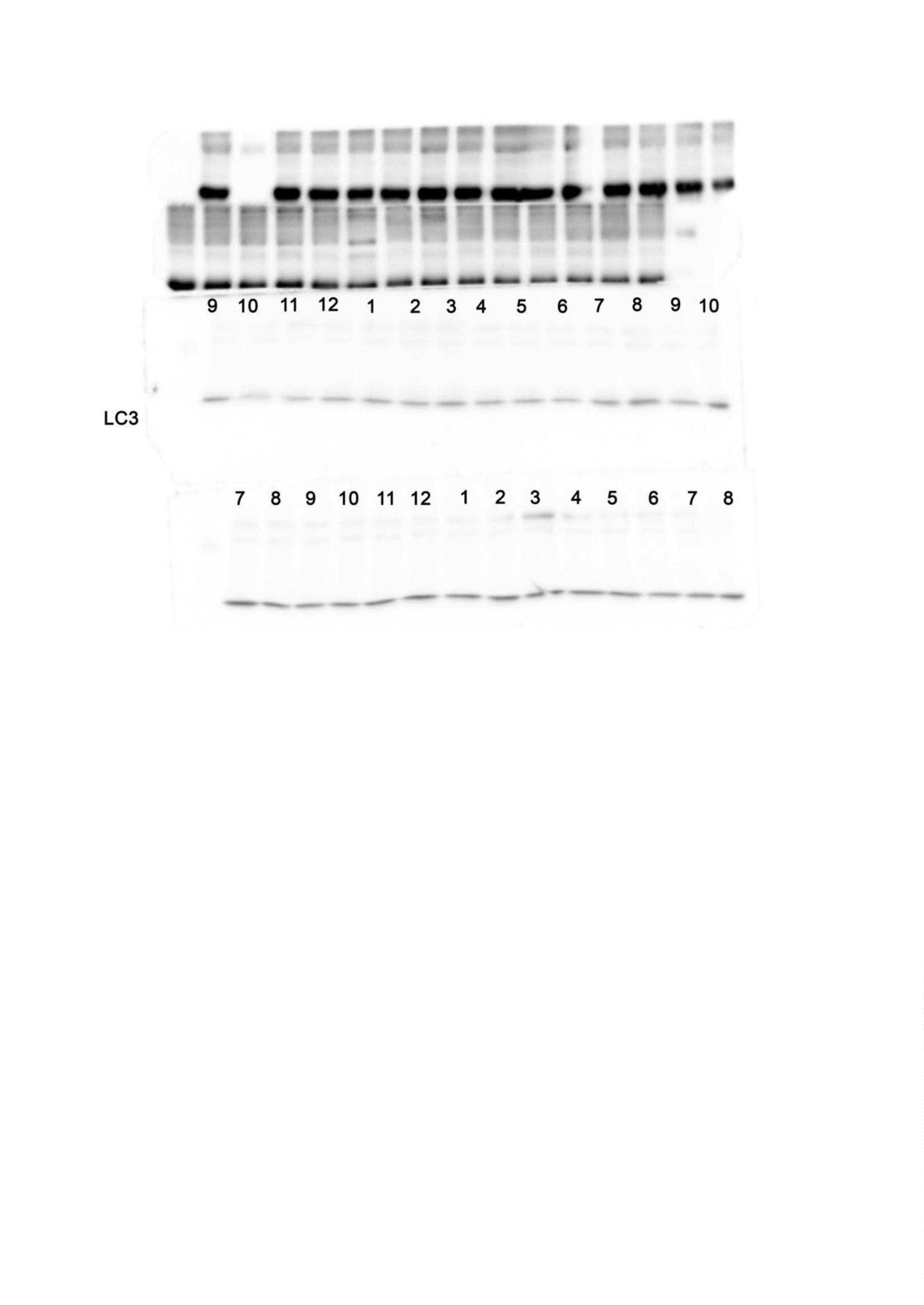

Supplement: Supplementary file 2 — Additional file 1. [file 12964_2023_1270_MOESM1_ESM.docx]
